# Surfing in the storm: how *Paraburkholderia xenovorans* thrives under stress during biodegradation of toxic aromatic compounds and other stressors

**DOI:** 10.1093/femsre/fuaf021

**Published:** 2025-05-19

**Authors:** Valentina Méndez, Mario Sepúlveda, Katherin Izquierdo-Fiallo, Constanza C Macaya, Teresa Esparza, Ximena Báez-Matus, Roberto E Durán, Gloria Levicán, Michael Seeger

**Affiliations:** Molecular Microbiology and Environmental Biotechnology, Department of Chemistry & Center of Biotechnology Daniel Alkalay Lowitt, Universidad Técnica Federico Santa María, Avenida España 1680, 2390123 Valparaíso, Chile; Millennium Nucleus Bioproducts, Genomics and Environmental Microbiology (BioGEM), Avenida España 1680, 2390123 Valparaíso, Chile; Molecular Microbiology and Environmental Biotechnology, Department of Chemistry & Center of Biotechnology Daniel Alkalay Lowitt, Universidad Técnica Federico Santa María, Avenida España 1680, 2390123 Valparaíso, Chile; Millennium Nucleus Bioproducts, Genomics and Environmental Microbiology (BioGEM), Avenida España 1680, 2390123 Valparaíso, Chile; Department of Biology, Faculty of Chemistry and Biology, Universidad de Santiago de Chile, Avenida Libertador Bernardo O'Higgins 3363, Santiago, Chile; Molecular Microbiology and Environmental Biotechnology, Department of Chemistry & Center of Biotechnology Daniel Alkalay Lowitt, Universidad Técnica Federico Santa María, Avenida España 1680, 2390123 Valparaíso, Chile; Millennium Nucleus Bioproducts, Genomics and Environmental Microbiology (BioGEM), Avenida España 1680, 2390123 Valparaíso, Chile; Molecular Microbiology and Environmental Biotechnology, Department of Chemistry & Center of Biotechnology Daniel Alkalay Lowitt, Universidad Técnica Federico Santa María, Avenida España 1680, 2390123 Valparaíso, Chile; Millennium Nucleus Bioproducts, Genomics and Environmental Microbiology (BioGEM), Avenida España 1680, 2390123 Valparaíso, Chile; Molecular Microbiology and Environmental Biotechnology, Department of Chemistry & Center of Biotechnology Daniel Alkalay Lowitt, Universidad Técnica Federico Santa María, Avenida España 1680, 2390123 Valparaíso, Chile; Millennium Nucleus Bioproducts, Genomics and Environmental Microbiology (BioGEM), Avenida España 1680, 2390123 Valparaíso, Chile; Molecular Microbiology and Environmental Biotechnology, Department of Chemistry & Center of Biotechnology Daniel Alkalay Lowitt, Universidad Técnica Federico Santa María, Avenida España 1680, 2390123 Valparaíso, Chile; Millennium Nucleus Bioproducts, Genomics and Environmental Microbiology (BioGEM), Avenida España 1680, 2390123 Valparaíso, Chile; Department of Biology, Faculty of Chemistry and Biology, Universidad de Santiago de Chile, Avenida Libertador Bernardo O'Higgins 3363, Santiago, Chile; Molecular Microbiology and Environmental Biotechnology, Department of Chemistry & Center of Biotechnology Daniel Alkalay Lowitt, Universidad Técnica Federico Santa María, Avenida España 1680, 2390123 Valparaíso, Chile; Millennium Nucleus Bioproducts, Genomics and Environmental Microbiology (BioGEM), Avenida España 1680, 2390123 Valparaíso, Chile

**Keywords:** proteostasis, chaperone, stress, *Paraburkholderia*, aromatic compound, adaptation

## Abstract

The adaptive mechanisms of Burkholderiales during the catabolism of aromatic compounds and abiotic stress are crucial for their fitness and performance. The aims of this report are to review the bacterial adaptation mechanisms to aromatic compounds, oxidative stress, and environmental stressful conditions, focusing on the model aromatic-degrading *Paraburkholderia xenovorans* LB400, other Burkholderiales, and relevant degrading bacteria. These mechanisms include (i) the stress response during aromatic degradation, (ii) the oxidative stress response to aromatic compounds, (iii) the metabolic adaptation to oxidative stress, (iv) the osmoadaptation to saline stress, (v) the synthesis of siderophore during iron limitation, (vi) the proteostasis network, which plays a crucial role in cellular function maintenance, and (vii) the modification of cellular membranes, morphology, and bacterial lifestyle. Remarkably, we include, for the first time, novel genomic analyses on proteostasis networks, carbon metabolism modulation, and the synthesis of stress-related molecules in *P. xenovorans*. We analyzed these metabolic features *in silico* to gain insights into the adaptive strategies of *P. xenovorans* to challenging environmental conditions. Understanding how to enhance bacterial stress responses can lead to the selection of more robust strains capable of thriving in polluted environments, which is critical for improving biodegradation and bioremediation strategies.

## Teaser

Novel insights of the stress adaptation mechanisms related to aromatic degradation and abiotic stressors in *Paraburkholderia xenovorans*

## Introduction

Strains of the genus *Paraburkholderia* are part of the *Burkholderia sensu lato* multigenus complex. Species of *Burkholderia sensu lato* are now classified within seven distinct genera, all of which were previously described under the *Burkholderia* genus (Sawana et al. 2014, Dobritsa and Samadpour 2019). *Paraburkholderia* genus mainly comprises environmental strains associated with plants, soil, fungi, and aquatic niches (Sawana et al. 2014). In contrast, the *Burkholderia sensu stricto* genus includes mostly clinical and phytopathogenic strains. *Caballeronia, Trinickia, Mycetohabitans, Robbsia*, and *Pararobbsia* genera were reclassified from the *Burkholderia* and *Paraburkholderia* genera. Strains belonging to *Paraburkholderia* generally possess large genomes (>7 Mbp), are metabolically versatile, and are capable of adapting to diverse adverse conditions (Chain et al. 2006, Pérez-Pantoja et al. 2012, Herpell et al. 2021, Rodríguez-Castro et al. 2024). Bacteria from the Burkholderiales order have an impressive catabolic potential revealed by the presence of a high number of aromatic degradative pathways encoded in their genomes (Pérez-Pantoja et al. 2012). These features are useful for the application of efficient bioremediation strategies.

*Paraburkholderia xenovorans* strains are mainly present in soil and the rhizosphere of grass plants (Chain et al. 2006). *Paraburkholderia xenovorans* LB400, a Burkholderiales species, is a model aromatic-degrading bacterium that degrades an unusually wide range of persistent organic pollutants (POPs), including aromatic compounds and polychlorobiphenyls (PCBs) (Seeger et al. 1999, 2001, 2003, Chain et al. 2006). Recently, it has been reported that *P. xenovorans* LB400 is also a plant growth-promoting bacterium (Vega-Celedón et al. 2024). *Paraburkholderia xenovorans* possesses over 30 catabolic pathways for the degradation of aromatic compounds, including 11 central and more than 20 peripheral pathways—one of the highest numbers reported among bacteria. *P. xenovorans* LB400 genome (9.73 Mbp) was sequenced and characterized by Chain et al. (2006) with two chromosomes (C1 and C2) and a megaplasmid, revealing high genomic plasticity and redundancy. According to evolutionary analyses, >20% of *P. xenovorans* genes were recently acquired by horizontal gene transfer, reflecting a niche-specialized genomic composition within the genus (Chain et al. 2006). Therefore, it is no surprise that this strain, isolated from a PCB-polluted landfill in New York state, hosts a wide repertoire of genes for metabolizing aromatic compounds and PCBs, and carries diverse stress response genes. Its genome encodes key enzymes involved in the degradation of aromatic compounds, such as monooxygenases, dioxygenases, and hydroxylases (Chain et al. 2006, Méndez et al. 2011, Chirino et al. 2013, Rodríguez-Castro et al. 2024). Functional redundancy of the *P. xenovorans* LB400 genome has been experimentally observed in the catabolism of aromatic compounds, such as the benzoate and hydroxyphenylacetate pathways (Denef et al. 2006, Méndez et al. 2011).

Bacterial strains from the Burkholderiales and Pseudomonadales orders have been models for the study of aromatic degradation (Olivera et al. 1998, Dinkla et al. 2001, Jiménez et al. 2002, Segura et al. 2005, Chain et al. 2006, de Lorenzo et al. 2024, Rodríguez-Castro et al. 2024). An initial activation is required to metabolize aromatic substrates, which often involves redox modifications, such as monooxygenation or dioxygenation, performed by Rieske nonheme iron oxygenases (Gibson and Parales 2000). Degradation intermediates of most aromatic compounds eventually funnel into the ß-ketoadipate pathway and ultimately, to the central carbon metabolism for energy production (Gibson and Parales 2000). However, the mechanism of action of oxygenases leads to the formation of reactive oxygen species (ROS), which increases when aromatic substrates do not fit properly in the active center of these enzymes (Imbeault et al. 2000, Patrauchan et al. 2008). Upregulation of general and oxidative stress proteins has been reported in *Paraburkholderia, Pseudomonas, Acinetobacter*, and *Bacillus* strains during aerobic aromatic catabolism or upon exposure to aromatic compounds (Tam et al. 2006, Agulló et al. 2007, Martínez et al. 2007, Pieper and Seeger 2008, 2017, Lin 2017, Méndez et al. 2022a, Rodríguez-Castro et al. 2024).

Exposure to environmental stress, such as aromatic compounds that are widely distributed in the environment, causes detriment in bacterial fitness. In addition, other sources of stress are present in polluted ecosystems, such as scarcity of nutrients and soil salinity that has been intensified by climate change (Atai et al. 2023). However, there is a significant gap in the current knowledge about stress responses in bacteria, especially strains from the Burkholderiales order, during biodegradation and other abiotic factors. Stress responses in members of the Burkholderiales order—relevant in biodegradation, agriculture, and pathogenesis—have received comparatively less attention in the context of biodegradation, leaving a significant gap in understanding their adaptations to the stress associated with the degradation of aromatic compounds and to abiotic stressors. Moreover, the interplay between biodegradation and diverse bacterial stress response mechanisms remains largely unexplored.

This review summarizes relevant hints on physiological responses of *P. xenovorans*, other Burkholderiales, and relevant degrading bacteria to toxic compounds such as aromatic molecules and PCBs, and adaptive mechanisms to counteract oxidative stress, nutrient scarcity, and high salinity conditions. In addition, novel genomic insights in *P. xenovorans* and other model bacteria focused on proteostasis network, reducing power regeneration pathways, and synthesis of osmoprotectants will be presented to further understand adaptation mechanisms upon stress, which are crucial to improving bioremediation processes.

Aromatic-degrading bacteria, especially Burkholderiales, exhibit complex proteostasis mechanisms that allow them to adapt to harsh environmental conditions, such as oxidative stress caused by redox imbalances. Exposure to aromatic compounds and toxic intermediates induces oxidative stress, triggering a cascade of metabolic adaptations to redirect energy production toward stress responses. These adaptations often include shifts in amino acid metabolism, protein turnover, and protein synthesis, ensuring the maintenance of cellular homeostasis. Additionally, bacterial membranes play a crucial role in the uptake and transport of aromatic compounds, serving as a first line of defense against their toxic effects. Together, these strategies enable bacteria to support their survival, fitness, and functionality while metabolizing toxic aromatic compounds.

## Stress response during aromatic degradation

In nature, microorganisms are exposed to environmental stress factors that activate specific stress responses. The presence in the environment of numerous aromatic compounds derived from petroleum and lignin components, plant exudates, aromatic amino acids, and xenobiotics contributes to the high diversity of ecological niches. Thus, xenobiotics are not the only aromatic compounds microorganisms encounter in the environment they inhabit. The presence of diverse aromatic compounds, such as benzoate, hydroxyphenylacetates, phenol, toluene, and (chloro)biphenyls, triggers a stress response in degrading bacteria (Lambert et al. 1997, Domínguez-Cuevas et al. 2006, Agulló et al. 2007, Ma et al. 2020, Rodríguez-Castro et al. 2024). Diverse aromatic compounds and their metabolic intermediates are highly toxic to cells (Sikkema et al. 1995, Blasco et al. 1997, Cámara et al. 2004, Chirino et al. 2013, Agulló et al. 2017). During aerobic bacterial aromatic degradation, an increase in ROS may cause significant protein damage, which can lead to protein unfolding and aggregation, resulting in the induction of molecular chaperones as protection strategies.

The analysis of 80 Burkholderiales genomes published by Pérez-Pantoja et al. (2012) revealed significant catabolic potential, highlighting the presence of central ring-cleavage pathways and peripheral pathways associated with the biodegradation of a wide range of aromatic compounds. The most common pathways identified include the protocatechuate, catechol, homogentisate, and phenylacetyl-CoA ring-cleavage pathways, present in at least 60% of the genomes analyzed. The metabolically versatile strain *Burkholderia* sp. K24 degrades monocyclic aromatic hydrocarbons, including aniline, benzene, toluene, and xylene (Lee et al. 2016). Proteomic analyses in *Burkholderia* sp. K24 showed the upregulation of GroEL and GroES, the universal stress protein A (UspA), and a DNA-binding protein as part of the adaptive stress response (Lee et al. 2016). Adaptation mechanisms of a *Burkholderia* sp. strain during phenol degradation involve the upregulation of genes encoding Hsps, cold-shock proteins (Csp), DNA repair proteins, antioxidant proteins, and proteins related to the Krebs cycle and oxidative phosphorylation (Ma et al. 2020). *Burkholderia* sp. was able to grow after prolonged exposure to high phenol concentrations (1500 mg/l), indicating a significant level of tolerance to phenol due to stress adaptation mechanisms, which is crucial for its survival in polluted environments. The upregulation of a universal stress protein (Usp) and two genes encoding for alkyl hydroperoxide reductase subunits C and D was observed during methyl parathion degradation by *B. cenocepacia* CEIB S5-2 (Ortiz-Hernández et al. 2021).

The study of the genome- and proteome-wide defenses against aromatic compounds toxicity in *P. xenovorans* strain LB400 shows the induction of the molecular chaperones and stress proteins, indicating that exposure to diverse aromatic compounds is stressful for the cells. The molecular chaperones DnaK and GroEL are induced during chlorobiphenyl and *p-*cymene degradation (Agulló et al. 2007, 2017), whereas DnaK and HtpG are induced by dead-end metabolites of the biphenyl upper pathway of 4-chlorobiphenyl (Martínez et al. 2007). Moreover, exposure to (chloro)biphenyls (biphenyl, 4-chlorobiphenyl), chlorobenzoates (2-chlorobenzoate, 4-chlorobenzoate), and *p*-cymene by *P. xenovorans* increases the expression of molecular chaperones GroEL, DnaK, HtpG, and ClpB (Martínez et al. 2007, Agulló et al. 2017). Proteomic studies showed the induction of the GroEL, GroES, and DnaK chaperones during growth of *P. xenovorans* in 3-hydroxyphenylacetate and 4-hydroxyphenylacetate (Méndez 2017, Rodríguez-Castro et al. 2024). The chaperones DnaK, DnaJ, Hsp33, HtpG, and ClpB were upregulated in *P. xenovorans* cells grown on benzoate (Denef et al. 2006). Overall, these results suggest that the induction of molecular chaperones contributes to stabilizing proteins affected by these aromatic compounds. The presence of aromatic compounds in the cell membrane and the cytoplasm may destabilize the three-dimensional structure of proteins (Segura et al. 2005). ClpB and DnaK play a key role in rescuing damaged proteins from large aggregates in a process dependent on adenosine triphosphate (ATP) (Lee et al. 2003). DnaK and HtpG prevent aggregation and refold misfolded and aggregated intracellular proteins under stress conditions (Castanié-Cornet et al. 2014, Wickner et al. 2021). The activity of HtpG increases under oxidative and thermal stress (Wickner et al. 2021). The complex formed by GroEL–GroES chaperones binds to peptides during synthesis on ribosomes, leading to the correct folding of the active site subunits (Goemans et al. 2018).

Relevant studies have reported specific stress responses during aromatic degradation in strains outside the Burkholderiales order. Stress response of *Pseudomonas putida* DOT-T1E in the presence of toluene includes the induction of GroES, CspA (Segura et al. 2005). Induction of GroEL, GroES, DnaK, DnaJ, GrpE, Lon protease, and other proteins is part of the adaptive response of *P. putida* KT2440 to toluene and *o*-xylene (Domínguez-Cuevas et al. 2006). The chaperones DnaK, GrpE, and Clp are induced in *R. jostii* RHA1 in the presence of aromatic compounds (Patrauchan et al. 2008, Costa et al. 2017). The induction of these chaperones reflects the presence of misfolding and aggregated proteins during exposure to aromatic compounds.

## Oxidative stress response to aromatic compounds

Oxidative stress is triggered when ROS exceeds antioxidant mechanisms within the cell (Imlay 2008; 2013). Superoxide, H_2_O_2_, and hydroxyl radicals are the most common oxidizing compounds produced when molecular oxygen gains single electrons (Imlay 2008). ROS can be in the environment as disinfectants and antimicrobials or may be produced by bacterial metabolism, and are toxic due to their ability to interact with macromolecules, causing protein carbonylation, lipid peroxidation, and DNA mutation (Arenas et al. 2011; Imlay 2013).

**Figure 1.**
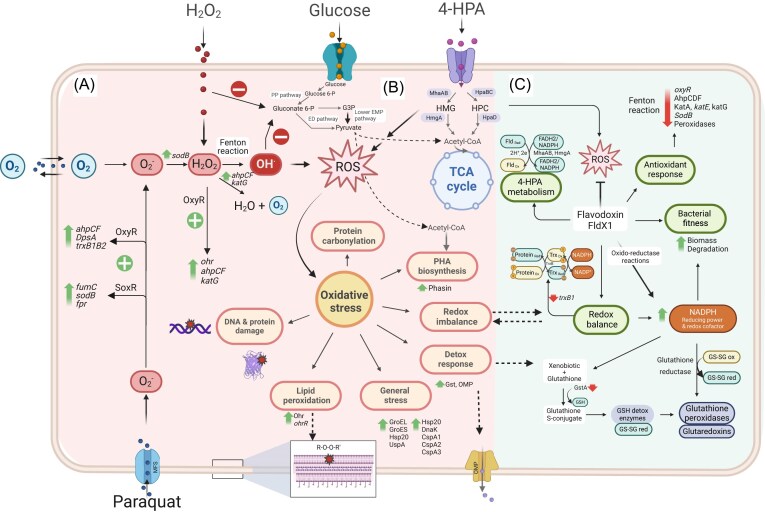
Molecular oxidative stress responses of *P. xenovorans* during exposure to oxidizing agents and 4‐hydroxyphenylacetate. (A) Response to paraquat and H_2_O_2_. (B) Response during 4‐hydroxyphenylacetate (4-HPA) catabolism in the presence of FldX1. 4‐HPA enters the cell through a transmembrane protein and is catabolized via the homoprotocatechuate and homogentisate degradation pathways. The preferred route, homogentisate, generates ROS via the MhaAB enzyme during an enzymatic reaction facilitated by FldX1, which serves as an electron shuttle. This is reflected by a downregulation of the antioxidant response (antioxidant proteins and genes), enhancing bacterial fitness. The long‐chain flavodoxin FldX1 acts as an electron shuttle between sources of reducing power and metabolic pathways. Green and red arrows indicate the upregulation and downregulation of genes, proteins, or metabolites, respectively. Created in https://BioRender.com.

ROS are generated during the aerobic metabolism of aromatic compounds in bacteria (Di Gennaro et al. 2011, Ponce et al. 2011, Agulló et al. 2017, Méndez 2017, Akkaya et al. 2018, Rodríguez-Castro et al. 2024), which leads to an oxidative stress response (Méndez et al. 2022a, Rodríguez-Castro et al. 2024). The oxidative stress response includes the induction of antioxidant proteins to restore redox homeostasis in the cell. The alkyl hydroperoxide reductase AhpC subunit is induced upon degradation of diverse aromatic substrates such as biphenyl, chlorobiphenyls, *p*-cymene, and 4-hydroxyphenylacetate in *P. xenovorans* (Agulló et al. 2007, Ponce et al. 2011, 2017, Méndez 2017, Rodríguez-Castro et al. 2024). Alkyl hydroperoxide reductase (AhpCF or AhpCD) detoxifies peroxides at low concentrations. Exposure of *P. xenovorans* cells to *p*-cymene showed the induction of the organic hydroperoxide resistance Ohr protein and the aconitase AcnA, whose role is to replace the function of aconitase AcnB under conditions of oxidative stress (Agulló et al. 2017). Exposure to 4‐hydroxyphenylacetate increases ROS formation in *P. xenovorans*. However, a protective role of the *P. xenovorans* long-chain flavodoxin FldX1 has been observed during growth on aromatic compounds. Overexpression of FldX1 improves the performance of *P. xenovorans* by enhancing its growth on 4‐hydroxyphenylacetate and degradation rates compared to the control strain (Rodríguez-Castro et al. 2024). Moreover, the downregulation of several enzymes involved in oxidative stress response was observed, such as Ohr, DpsA, KatE and SodB, GstA, and TrxB, suggesting that the flavodoxin FldX1 protects against oxidative stress (Rodríguez-Castro et al. 2024). In addition, an increased 4‐hydroxyphenylacetate degradation was observed in soil microcosms by *P. xenovorans* overexpressing FldX1 (Rodríguez-Castro et al. 2024). These results suggest a key role of the long-chain flavodoxin FldX1 in improving tolerance to oxidative stress triggered by these oxidizing agents and aromatic compounds (Fig. 1).

Proteome studies in *P. xenovorans* strain LB400 revealed the upregulation of oxidative stress response proteins during growth on phenylacetate. Two peroxidases (BxeA3905 and BxeA0528) and an uncharacterized stress-induced protein (BxeA0904) were significantly more abundant in the phenylacetate proteome compared to succinate-grown cells (Patrauchan et al. 2011). Interestingly, growth on phenylacetate, benzoate, and biphenyl induced a similar set of stress response proteins and genes involved in fatty acid metabolism (Denef et al. 2006, Patrauchan et al. 2011). Microarray experiments revealed the upregulation of seven catalases and the *oxyR* regulatory gene during growth on biphenyl (Denef et al. 2006). These results indicate a strong oxidative stress response in *P. xenovorans* grown on aromatic compounds.

Oxidative stress response has been observed in other aromatic-degrading bacteria. Proteomic studies on the Pseudomonadales strain *Acinetobacter calcoaceticus* during the degradation of catechol revealed a higher expression of the oxidative stress proteins AhpC and AhpF, and significantly higher catalase activity (Benndorf et al. 2001). *Acinetobacter calcoaceticus* grown on crude oil increases superoxide dismutase (SOD) levels (Sazykin et al. 2019).

The stress response has also been studied in model aromatic-degrading strains of the *Rhodococcus* genus. An antioxidant response was described in *R. jostii* RHA1, a potent PCB-degrader, showing the upregulation in presence of biphenyl, ethylbenzene, or benzene of catalase, AhpC, Hsp100 proteins, ATP-dependent Clp protease, and fatty acid desaturase (Gonçalves et al. 2006, Patrauchan et al. 2008). Proteomic studies evaluating oxidative stress in *R. jostii* RHA1 identified a broad range of upregulated proteins in the presence of the aromatic compound paraquat, including AhpC, AhpF, catalase, chaperones DnaK and GrpE, glutaredoxin, and glutathione peroxidase, suggesting a robust stress response (Costa et al. 2017).

An upregulation of genes involved in oxidative stress response was observed in *Rhodococcus aetherivorans* I24 in the presence of PCBs (Puglisi et al. 2010). These include the *groEL, ahpC, sodA* (SOD), *katG* (catalase/peroxidase), and *trxB* (thioredoxin reductase) genes. Upregulated catabolic genes include those encoding the ring-hydroxylating dioxygenase BhpC, catechol 2,3-dioxygenase, carveol dehydrogenase, phenol hydrolase, and transporter genes. However, the genes *bphAaAbAcAd* of the biphenyl pathway were not upregulated. *Rhodococcus erythropolis* in presence of cyclohexane, naphthalene, and diesel increases the expression of genes encoding the cytochrome P450 and the Fe/Mn SODs, indicating an oxidative stress response to hydrocarbons (Sazykin et al. 2019).

### Oxidative stress response is regulated by OxyR and SoxR

The oxidative stress response in bacteria is primarily controlled by the transcriptional regulators OxyR and SoxR, which can sense ROS and trigger a specific response. The OxyR and SoxR mechanisms have been well-studied in *Escherichia coli*. Inside the cells, the presence of H_2_O_2_ is sensed by OxyR. OxyR belongs to the family of LysR regulators and acts as a transcriptional activator of oxidative stress genes. In the presence of H_2_O_2_, *E. coli* OxyR binds as a tetramer near the –35 region of at least 20 oxidative stress genes, activating them at the transcriptional level. Some of these include the genes *dpsA* (DNA and iron-binding protein), *gorA* (GSH reductase), *grxA* (glutaredoxin), *katG*, and *ahpCF* (Imlay 2008). These enzymes are essential for the survival of bacteria under oxidative stress. Previous studies have shown the presence of a remarkable number of stress-related genes in the genome of *P. xenovorans* LB400, revealing a robust set of genes for oxidative stress response (Méndez 2017, Méndez et al. 2022a). OxyR regulon-related genes identified in *P. xenovorans* include *ahpC* (alkyl hydroperoxide reductase subunit C; two copies), *ahpF* (alkyl hydroperoxide reductase subunit F; nine copies), *kat* (catalase; six copies), *gorA* (glutathione peroxidase; one copy), *dpsA* (one copy), *trxB* (thioredoxin reductase; one copy), and *fur* (ferric uptake regulator; one copy) (Méndez 2017, Méndez et al. 2022a). Other stress-related genes in *P. xenovorans* include *fumC* (fumarate hydratase; one copy), *acn* (aconitate hydratase; three copies), *sodB* (Cu–Zn and Mn–Fe SODs; four copies), *fpr* (NADP-dependent ferredoxin reductase; one copy), and *fldX* (flavodoxin X; two copies) (Méndez 2017, Méndez et al. 2022a). These genes are regulated by SoxRS in *E. coli*, which are activated by superoxide (Imlay 2008).

SODs are enzymes that catalyze the conversion of superoxide into H_2_O_2_, while catalases and peroxidases are present in the cytoplasm and generate H_2_O and O_2_ from H_2_O_2_. SODs are considered the first defense involved in superoxide detoxification (Imlay 2008). In *E. coli*, SodC (Cu) is in the periplasm, whereas SodA (Mn) and SodB (Fe) are cytoplasmic or cytoplasmic/membrane-bound enzymes. The SoxRS regulon in *E. coli* activates the transcription of *sodA* (Mn SOD), *zwf* (glucose-6-phosphate dehydrogenase), *fldA* and *fldB* (flavodoxins A and B), *fur* (ferric uptake regulator), *nfo* (DNA repair), *micF* (small RNA that negatively regulates the expression of porin F), and other genes in response to superoxide-generating compounds, such as the herbicide paraquat (Pomposiello and Demple 2001, Imlay 2008). However, these models differ from those described in nonenterobacteria. The *soxS* gene, a DNA-binding transcriptional regulator in *E. coli*, is absent in nonenterobacteria, suggesting alternative regulation roles of the SoxR regulon. For example, in the nonenterobacterium *Pseudomonas aeruginosa* PAO1, which also has a wide repertoire of oxidative stress response genes activated by OxyR, this is activated in the presence of H_2_O_2_ and paraquat (Ochsner et al. 2000). In *P. aeruginosa* PAO1, SoxR activates monooxygenase and transporter genes that may be involved in synthesizing phenazines (Dietrich et al. 2008). In *P. xenovorans*, the OxyR transcriptional regulator has a key protective role against H_2_O_2_ and paraquat (Méndez et al. 2022a). Unlike the separate OxyR- and SoxRS-mediated responses triggered by H_2_O_2_ and superoxide in *E. coli*, respectively, this study reveals a broad stress response in *P. xenovorans* that is primarily regulated by OxyR. Recent studies in *P. xenovorans* revealed relevant hints on its response to oxidizing compounds at the proteome and transcriptional levels. Growth, susceptibility, and ROS formation assays in *P. xenovorans* cells reveal a higher sensitivity to paraquat than H_2_O_2_. The herbicide paraquat is a widely studied oxidizing agent due to the redox cycling reactions that produce ROS (Méndez et al. 2022a). Transcriptional analyses showed in *P. xenovorans* cells, upon exposure to H_2_O_2_, the upregulation of the *oxyR, ahpC1, katE*, and *ohrB* genes. In addition, the *oxyR, fumC, ahpC1, sodB1*, and *ohrB* genes were induced in the presence of paraquat (Méndez et al. 2022a). Proteome analysis revealed that the herbicide paraquat induces oxidative stress response proteins, such as AhpCF, DpsA, the universal stress protein UspA, and the RNA chaperone CspA. Paraquat and H_2_O_2_ induced the Ohr protein, which is involved in organic peroxide resistance. Notably, the overexpression of the *oxyR* gene in *P. xenovorans* significantly decreased ROS formation and the susceptibility to paraquat, suggesting a broad antioxidant response regulated by OxyR (Méndez et al. 2022a) (Fig. 1).

Induction of the electron shuttle flavodoxin is another common feature of the antioxidant response for cell redox balance. Flavodoxins are small electron transfer flavoproteins, highly isofunctional with ferredoxins, expressed under oxidative stress and iron limitation (Sancho 2006, González and Fillat 2018). Remarkably, overexpression of the long-chain flavodoxin IsiB from the cyanobacterium *Anabaena* sp. PCC7119 in tobacco plants confers resistance to the redox-cycling herbicide paraquat and enhances the biodegradation of 2,4-dinitrotoluene (Tognetti et al. 2007). In addition, overexpression of the flavodoxin FldP protects *P. aeruginosa* from oxidative stress, decreasing H_2_O_2_-induced cell death and DNA hypermutability (Moyano et al. 2014). Similarly, previous reports showed that overexpression of the *P. xenovorans* long-chain flavodoxin FldX1 exerts a protective role against stress induced by paraquat and H_2_O_2_ (Rodríguez-Castro et al. 2019)_._ The strain overexpressing FldX1 showed a notable increase in survival (>70%) after exposure to the herbicide paraquat, suggesting that FldX1 may enhance tolerance to paraquat but not to H_2_O_2_ under specific conditions. FldX1 recombinant cells show lower lipid peroxidation after exposure to paraquat and reduced protein carbonylation after H_2_O_2_ exposure, compared to the control strain. A downregulation of several oxidative stress-related genes is observed (*katG, hpf, trxB1*, and *ohr*) after exposure to paraquat, suggesting a specific regulatory response to oxidative stress (Rodríguez-Castro et al. 2019) (Fig. 1).

### Effects of toxic metabolites

Production of unstable intermediates during dioxygenation reactions in aromatic catabolism can also increase oxidative stress, leading to cellular damage (Nojiri et al. 2001). In *P. xenovorans*, formation of toxic metabolic intermediates during aromatic degradation pathways has been reported. For example, the antibiotic picolinic acid is produced during 2-aminophenol degradation, which has an inhibitory effect on *P. xenovorans* growth (Chirino et al. 2013). The well-known antibiotic picolinic acid may be produced by a spontaneous nonenzymatic reaction from 2-aminomuconate-6-semialdehyde, which is the reaction product of the 2-aminophenol-1,6-dioxygenase (Chirino et al. 2013).

**Figure 2.**
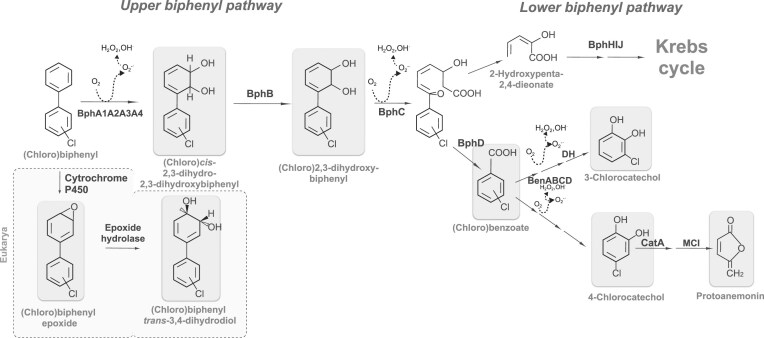
(Chloro)biphenyl, benzoate, and catechol catabolic pathways and formation of toxic metabolic intermediates. The enzymes of the upper (chloro)biphenyl pathway are encoded by the *bph* locus of *P. xenovorans* LB400. Toxic intermediates are shown in gray: (chloro)2,3-dihydro-2,3-dihydroxybiphenyl and (chloro)2,3-dihydroxybiphenyl. Enzymes: BphA1A2A3A4, biphenyl-2,3-dioxygenase; BphB, 2,3-dihydro-2,3-dihydroxybiphenyl-2,3-dehydrogenase; BphC, *cis*-2,3-dihydrobiphenyl-2,3-diol dehydrogenase; BphD, 2-Hydroxy-6-oxo-6-phenylhexa-2,4-dienoate hydrolase; BphH, 2-hydroxypenta-2,4-dienoate hydratase; BphI, 4-hydroxy-2-oxovalerate aldolase; BphJ, acetaldehyde dehydrogenase; BenA, benzoate 1,2-dioxygenase α subunit; BenB, benzoate 1,2-dioxygenase ß subunit; BenC, benzoate 1,2-dioxygenase ferredoxin reductase component; BenD, benzoate diol dehydrogenase; DH, dehydrogenase; CatA, catechol 1,2 dioxygenase; and MCI, muconate cycloisomerase. Formation of ROS by BphA1A2A3A4 and BphC is indicated.

PCB degradation by *P. xenovorans* and other bacteria is often incomplete, with a concomitant accumulation of metabolic intermediates. Specific PCB congeners and chlorobenzoates, which accumulate during PCB degradation, significantly reduce the viability of bacterial cells (Cámara et al. 2004). 4-Chlorobenzoate and 2-chlorobenzoate inhibit *P. xenovorans* growth on glucose. Moreover, *P. xenovorans*-exposed cells to 4-chlorobenzoate show an increased number and size of electron-dense granules in the cytoplasm, which may be polyphosphates (Martínez et al. 2007) that have been linked to stress protection mechanisms, serving as scaffolds to stabilize protein structure (Gray et al. 2014). Polyphosphates are stress-resistant nonproteinaceous chaperones that bind to soluble unfolded proteins, maintaining them in a folding-competent conformation (Reichmann et al. 2018). (Chloro)biphenyls differ in their toxicity compared with the biotransformation products. (Chloro)dihydrodiols and (chloro)dihydroxybiphenyls are highly toxic metabolites, affecting cell viability significantly more than (chloro)biphenyls. Partial degradation of PCBs by the enzymes BphA and BphB produces toxic metabolic intermediates. (Chloro)2,3-dihydroxybiphenyl drastically decreases the cell viability of *P. xenovorans* (Seeger et al. 1995, 1999, Seah et al. 2000, Cámara et al. 2004). Figure 2 shows the formation of the toxic metabolites (chloro)dihydrodiol and (chloro)dihydroxybiphenyl of the upper biphenyl pathway in *P. xenovorans*. Hydroxylated metabolites of PCBs can affect bacterial DNA content, inhibiting cell separation (Hiraoka et al. 2002). The toxicity of metabolites generated during the oxidation of PCBs may partly explain the recalcitrance to the biodegradation of these pollutants. Biotransformation of PCBs into more toxic derivatives is a well-documented phenomenon analogous to the activation in higher organisms of xenobiotics and drugs. For example, the oxidation of specific compounds by cytochrome P450 generates cytotoxic or carcinogenic products (Fig. 2). Cytochrome P450 activates carcinogenic compounds, producing electrophilic intermediates that can bind to DNA, leading to mutations and cellular transformation associated with cancer development (Abu-Bakar et al. 2022). Conversely, the induction of chloroacetaldehyde dehydrogenase has been associated with a decrease in the toxic chlorinated aliphatic compounds resulting from PCB degradation (Denef et al. 2005). In this context, cellular mechanisms provide a defense against these toxic aromatic metabolites. For example, BphK is a glutathione *S*-transferase that occurs in diverse biphenyl pathways (Bartels et al. 1999). BphK catalyzes the dehalogenation of 3-chloro-2-hydroxy-6-oxo-6-phenyl-2,4-dieneoates, compounds that are produced by the metabolism of PCBs by the catabolic enzymes BphA, BphB, and BphC (Fortin et al. 2005), which inhibit the next enzyme, BphD. BphK is also able to dehalogenate 4-chlorobenzoate, the product of 4-chlorobiphenyl degradation (Gilmartin et al. 2003).

Furthermore, chlorobenzoates, which are often dead-end products in bacterial PCB metabolism, can be converted into harmful downstream compounds such as chlorocatechols. 3-Chlorocatechol can inactivate extradiol dioxygenases, such as the 2,3-dihydroxybiphenyl 1,2-dioxygenase, therefore disrupting the upper biphenyl degradation pathway (Vaillancourt et al. 2002). Funneling of 4-chlorocatechol into the widespread ß-ketoadipate pathway can result in the production of the antibiotic protoanemonin (Blasco et al. 1995) (Fig. 2), which has been suggested to decrease the survival of PCB-degrading organisms in soil microcosm studies (Blasco et al. 1997).

Studies using the PCB-degrading strains *P. xenovorans* and *R. jostii* RHA1 also demonstrated that the toxicity of PCBs arises mainly from the production of harmful metabolites during the degradation (Seeger 1996, Parnell et al. 2006). While PCBs are associated with the cell membrane fraction, no significant effects on bacterial viability or growth rate were observed under nondegrading conditions. However, significant strain-dependent differences emerged when cells metabolized PCBs, with *P. xenovorans* showing high tolerance to PCB degradation-related toxicity, whereas strain RHA1 was highly sensitive (Parnell et al. 2006).

## Metabolic adaptation to oxidative stress

Aromatic-degrading bacteria such as *P. xenovorans* LB400 and *P. putida* KT2440 possess strong NADPH-consuming antioxidative systems that deal with ROS accumulation during the catabolism of aromatic compounds (Ponce et al. 2011, Rodríguez-Castro et al. 2019, Nikel et al. 2021). Therefore, maintaining a high reducing power pool (NADPH/NADP^+^ ratio) by rerouting carbon source metabolization contributes to ROS detoxification by key antioxidant systems, such as glutathione, thioredoxins, and alkyl hydroperoxide reductases (Pastor et al. 2019, Nikel et al. 2021). The most common mechanism to manage oxidative stress involves the action of ROS-detoxifying enzymes such as catalases, peroxidases, and hydroperoxide reductases. The corresponding reactions consume metabolic NADPH, which provides the reductive power to counteract the toxic effects of ROS via reduced glutathione (Tamburro et al. 2004). For example, the glutathione cycle, which connects the reduced (GSH) and the oxidized (GS–SG) forms of the thiol, is a preferred reductant of ROS via the glutathione peroxidase and glutaredoxin enzymes. Thus, the reduced glutathione pool is restored by using NADPH as a reductant (Vašková et al. 2023).

*Pseudomonas aeruginosa* PAO1 and other pseudomonads catabolize glucose predominantly through the Entner–Doudoroff (ED) pathway, which is extremely efficient in generating the NADPH required for the function of several antioxidant responses with low protein expenses (Chavarría et al. 2013, Berger et al. 2014). Similarly, *P. aeruginosa* can adapt and arrange central metabolism to efficiently utilize different carbon sources, maintaining NADPH and anabolism, allowing it to survive under various environmental conditions (Dolan et al. 2020). An increase in the NADPH pool provides robust machinery for metabolizing novel xenobiotic substrates in *P. putida* (Akkaya et al. 2018).

**Figure 3.**
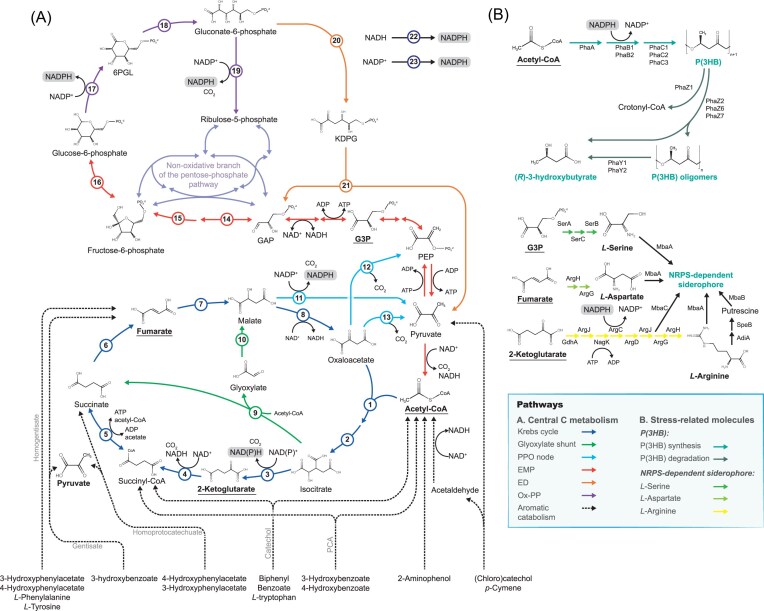
Central carbon metabolism and metabolic pathways for stress-related molecules in *P. xenovorans* LB400. Pathways are shown for: (A) catabolism of aromatic compounds, and (B) metabolism of stress-related molecules. Intermediates in bold are anabolic precursors for the synthesis of stress-related molecules. 1, Citrate synthase (GltA1 and GltA2). 2, Aconitate hydratase (AcnA1 and AcnA2). 3, Isocitrate dehydrogenase (Idh1, Idh2, and Idh3). 4, 2-Ketoglutarate dehydrogenase (SucA). 5, Succinyl-CoA synthase (SucC). 6, Succinate dehydrogenase (SdhC). 7, Fumarate hydratase (FumCB). 8, Malate dehydrogenase (Mdh). 9, Isocitrate lyase (MtbI). 10, Malate synthase (AceB or GlcG). 11, Malate dehydrogenase (oxaloacetate decarboxylating) (MaeB1, MaeB2, and MaeB3). 12, Oxaloacetate decarboxylase (Oad). 13, Phosphoenolpyruvate (PEP) carboxykinase (PckG). 14, Fructose-1,6-biphosphate (FBP) aldolase (CbbA1, CbbA2, and CbbA3). 15, FBP 1,6-biphosphatase (Fbp1 and Fbp2). 16, Glucose-6-phosphate isomerase (Pgi). 17, Glucose-6-phosphate dehydrogenase (Zwf1, Zwf2, and Zwf3). 18, Phosphogluconolactonase (Pgl). 19, 6-Phosphogluconate dehydrogenase (Pgdh1 and Pgdh2). 20, 6-Phosphogluconate dehydratase (Edd). 21, 2-Keto-3-deoxy-6-phosphogluconate (KDPG) aldolase (Eda). 22, Proton translocating transhydrogenase (PntAB). 23, NADP-dependent ferredoxin oxidoreductase (Fpr). G3P, glycerate-3-phosphate. GAP, glyceraldehyde-3-phosphate. 6-PGL, 6-phosphogluconolactone. PhaZ, poly(3-hydroxybutyrate) (P(3HB)) depolymerase. PhaY, P(3HB) oligomer hydrolase. P(3HB), poly(3-hydroxybutyrate). **(*R*)**-3HB, **(*R*)**-3-hydroxybutyrate. NRPS, nonribosomal peptide synthase. SerA, d-3-phosphoglycerate dehydrogenase. SerC, phosphoserine aminotransferase. SerB, phosphoserine phosphatase. GdhA, l-glutamate dehydrogenase. ArgJ, arginine synthesis bifunctional protein. NagK, acetylglutamate kinase. ArgC, *N*-acetyl-γ-glutamylphosphate reductase. ArgD, acetylornithine aminotransferase. ArgG, argininosuccinate synthase. ArgH, argininosuccinate lyase. AdiA, Arginine decarboxylase. SpeB, agmatinase. MbaA, MbaB, nonribosomal peptide synthases (NRPS). MbaC, l-ornithine-5-monooxygenase (Vargas-Straube et al. 2016). PhaA, 2-ketothiolase. PhaB, **(*R*)**-3-hydroxybutyryl-CoA reductase. PhaC, polyhydroxyalkanoate (PHA) synthase (Urtuvia et al. 2018). PPO, PEP, pyruvate, oxaloacetate node. EMP, Embden–Meyerhof–Parnas. ED, Entner–Doudoroff. Non-OxPP, the nonoxidative branch of pentose–phosphate (PP) pathway. Ox-PP, oxidative branch of the PP pathway. NADPH formation is highlighted in the corresponding enzymatic reactions. Central metabolic pathways were evaluated in *P. xenovorans* LB400 through the BlastKO software of the Kyoto Encyclopedia of Genes and Genomes (KEGG) database. Enzymes were corroborated through the bidirectional best-hit approach using the BlastP tool against the Swiss-Prot curated database considering sequences identities >30% and alignment coverage >70% of the query sequence. Sequences with empirical evidence at the transcript or protein level were considered as references. Gene context was also evaluated with the KEGG genome database.

Genome-guided metabolic reconstruction revealed that in *P. xenovorans*, sugars are metabolized via the ED, pentose phosphate (PP), and lower Embden–Meyerhof–Parnas (EMP) pathways, which produce more reducing power through NADPH synthesis than the complete EMP glycolysis (Álvarez-Santullano et al. 2021) (Fig. 3). Furthermore, *P. xenovorans* LB400 exhibits higher gene redundancy in carbohydrate and fatty acid metabolism compared to other *Burkholderia sensu lato* strains, suggesting higher robustness and versatility (Álvarez-Santullano et al. 2021). These metabolic networks allow the adaptation under the changing environmental conditions where species of *Paraburkholderia* and *Pseudomonas* strains inhabit.

During aromatic metabolism, *P. xenovorans* channels the degradation products into the central carbon metabolism through the Krebs cycle and the gluconeogenic pathways to produce reductive power and anabolic precursors (Fig. 3A). Central carbon metabolism in *P. xenovorans* possesses high gene redundancy that confers robustness against stress. For example, the ROS-sensitive [4Fe-4S]-dependent aconitase (BxeB2903) is downregulated in *P. xenovorans* exposed to the herbicide paraquat, upregulating the oxidation-resistant enzymes aconitase (BxeB1533) and fumarase hydratase FumC (BxeA1038) (Méndez et al. 2022a).

On the other hand, *P. xenovorans* possesses an incomplete glycolytic EMP pathway due to the absence of phosphofructokinase (Pfk). Therefore, glycolysis occurs through the ED and PP pathways (Fig. 3A). The incomplete glycolytic EMP pathway has been described in *Pseudomonas* and *Chromohalobacter* species, while a cyclic arrangement among the PP, ED, and gluconeogenic EMP pathways efficiently produces NADPH to counteract ROS (Pastor et al. 2019, Nikel et al. 2021, Wilkes et al. 2023). The incomplete glycolytic EMP is common in *Paraburkholderia* and *Caballeronia* species and to a lower extent in *Burkholderia* species (Álvarez-Santullano et al. 2021).

### Genomic analysis of NADPH regenerative pathways in *P. xenovorans*

The cofactor NADPH is an important source of reductive power to counteract endogenous oxidative stress and also participates in the anabolism of biological molecules (Fig. 3B). The reduction of NADP^+^ into NADPH can be conducted by the central carbon metabolism enzymes, such as glucose-6-phosphate dehydrogenase (Zwf), phosphogluconate dehydrogenase (Pgdh), isocitrate dehydrogenase (Idh), malic enzyme (MaeB), and malate dehydrogenase (MdhB). In *P. xenovorans*, the genome harbors a notably high copy number of genes encoding the NADPH-regenerating enzymes Zwf, Pgdh, MaeB, MdhB, and Idh, relative to other central carbon metabolism enzymes (Table S2). This genetic redundancy, an attribute of strain LB400, correlates with its metabolic plasticity and robustness in modulating intracellular NADPH levels. In *P. xenovorans* LB400, the Zwf enzymes are encoded by the *zwf1* (BxeA3452), *zwf2* (BxeB0215), and *zwf3* (BxeB1764) genes, whose genomic contexts are related to the central carbon metabolism, storage polymer accumulation (e.g. glycogen and polyphosphates), and serine synthesis, respectively. These genomic features may be a trait for adapting to nutrient-deprived environments or to fulfill anabolic demands (Álvarez-Santullano et al. 2021). The redundancy of Zwf isoenzymes correlates with the presence of the ED pathway as the unique glycolytic strategy, in contrast to organisms utilizing the EMP pathway. *Pseudomonas putida* KT2440, which utilizes the ED pathway, encodes three Zwf isoforms with distinct NAD(P)^+^ cofactor specificities (Volke et al. 2021), potentially conferring metabolic flexibility to modulate the cellular redox balance under stress conditions. Similarly, the *idh1* (BxeA0797) and *idh2* (BxeB0532) genes encode NADP^+^-dependent Idh enzymes, while the *idh3* gene (BxeC0665) encodes a NAD^+^-dependent enzyme, which may be useful to modulate the intracellular NADPH/NADH ratio according to cellular requirements. Interestingly, the NADP^+^-dependent Idh enzyme is significantly more abundant in *P. xenovorans* cells grown on benzoate compared with succinate-grown cells (Denef et al. 2005). In addition, *P. xenovorans* harbors three NADP^+^-dependent *maeB* genes that encode the NADP^+^-dependent malic enzyme. A gene copy (BxeA0283) is located next to the *fldx1* gene that plays an important role by increasing reducing power during growth on hydroxyphenylacetates (Rodríguez-Castro et al. 2019, 2024). Another redox-regulating mechanism is the reduction of NADP^+^ using NADH as a cofactor by the transhydrogenase enzyme PntAB, which is also redundant in *P. xenovorans* (Table S2) (Spaans et al. 2015). The *pntAB* gene (BxeA4006) is located upstream of a paraquat-inducible transporter (Nakayama and Zhang-Akiyama 2017), showing a favorable genetic context for response to toxic compounds. An alternative source of NADPH in *P. xenovorans* is the ferredoxin NADPH oxidoreductase Fpr, encoded by the BxeA4345 gene, which possesses a 63% amino acid identity with the Fpr enzyme of *Azotobacter vinelandii* OP1 that reduces NADP^+^ and oxidizes NADPH (Fig. 3A) according to the intracellular redox conditions (Sridhar et al. 1998).

### Polyhydroxyalkanoate synthesis as an adaptive strategy

In response to nutrient imbalance (e.g. carbon excess and nitrogen or phosphorus scarcity), bacteria and archaea modify their metabolism to synthesize and accumulate different biomolecules, such as polyhydroxyalkanoates (PHAs), triacylglycerols, wax esters, and polyphosphates (Urtuvia et al. 2018, Obruca et al. 2021, Pátek et al. 2021, Srivastava et al. 2022, Ben Abdallah et al. 2025). Exogenous stresses, such as nutrient scarcity, heat shock, and high concentration of heavy metals, can be mitigated through the chaperone effects of intracellular PHA granules, phasin proteins (PhaPs), and the PHA degradation products (*R*)-3-hydroxyacyl oligomers (Müller-Santos et al. 2021). Interestingly, the induction of PhaPs during the growth of *P. xenovorans* LB400 on biphenyl has been observed (Denef et al. 2005). A repertoire of five PhaPs has been described in *P. xenovorans* strain LB400, including PhaP1 (BxeA1544), PhaP2 (BxeA1874), PhaP3 (BxeB0319), PhaP4 (BxeB0720), and PhaP5 (BxeB2336) (Urtuvia et al. 2018). The PhaP1 phasin is induced by the ROS-generating herbicide paraquat (Méndez et al. 2022a). On the other hand, under a carbon excess and nitrogen starvation, intracellular poly(3-hydroxybutyrate) (P(3HB)), a type of PHA, in *P. xenovorans* accumulates, upregulating the PHA synthase (*phaC1*; BxeA2343) and the PHA depolymerase (*phaZ1*; BxeA3308) genes (Urtuvia et al. 2018). The PhaC enzyme catalyzes the last step of PHB polymerization, while the PhaZ1 degrades P(3HB) oligomers into (*R*)-3-hydroxybutyrate ((*R*)-3HB), suggesting that P(3HB) mobilization is an adaptive strategy during nutrient scarcity stress. Interestingly, the P(3HB) degradation product (*R*)-3HB and derived compounds scavenge hydroxyl radicals, exerting alternative protection against ROS in bacterial cells challenged by different stress sources (Obruca et al. 2016, 2020, Müller-Santos et al. 2021).

Synthesis of P(3HB) has been reported in *P. xenovorans* grown under high carbon concentration (glucose, mannitol, or xylose) and nitrogen-limiting conditions (Urtuvia et al. 2014, 2018), a significant source of exogenous stress. *P. xenovorans* possesses complete anabolic pathways and highly redundant NADPH regeneration pathways to synthesize compounds (e.g. PHAs and siderophores) from central carbon intermediates that may be obtained from the catabolism of carbohydrates or aromatic compounds (Fig. 3). In addition, *P. xenovorans* possesses the anabolic pathways to obtain other PHAs from gluconate, fructose, glycerol, arabinose, and fatty acids (Acevedo et al. 2018, Urtuvia et al. 2018, Álvarez-Santullano et al. 2021).

Synthesis of P(3HB) starts from two acetyl-CoA that are converted by the 3-ketothiolase PhaA into acetoacetyl-CoA, which is reduced into the precursor (*R*)-3-hydroxybutyryl-CoA ((*R*)-3HB-CoA) through the NADPH-dependent acetoacetyl-CoA reductase PhaB (Fig. 3C). NADPH utilization as a cofactor by the PhaB enzyme places PHAs synthesis as a reductive power sink or as a “pseudo fermentation” (Obruca et al. 2020, Velázquez-Sánchez et al. 2020). (*R*)-3HB-CoA is finally polymerized by PHA synthases (PhaC) into P(3HB) (Fig. 3C).

*Paraburkholderia xenovorans* synthesizes PHAs from the β-oxidation intermediates *trans*-enoyl-CoA and (*S*)-3-hydroxyacyl-CoA, catalyzed by the *R*-specific enoyl-CoA hydratase PhaJ (BxeB0140) and the epimerase activity of a (*S*)-3-hydroxyacyl-CoA dehydrogenase multienzyme complex FadB (BxeC0280) into (*R*)-3-hydroxyacyl-CoA ((*R*)-3HA-CoA). (*R*)-3HA-CoA can be further polymerized into PHA. An additional 3-ketothiolase enzyme (BktB) encoded by BxeA2335 in *P. xenovorans* yields ketovaleryl-CoA from propionate-CoA and acetyl-CoA, which can be further reduced into the PHA precursor (*R*)-3-hydroxyvaleryl-CoA to obtain the poly(3-hydroxybutyrate-*co*-3-hydroxyvalerate) copolymers. Overall, the type of PHA depends on the chain length of the acyl moiety of the (*R*)-3HA-CoA precursor and the PHA synthase class. *Paraburkholderia xenovorans* possesses the BxeA2343 gene encoding a class I PHA synthase PhaC1, which is located in the *phaC1ABR* gene cluster upstream of the *bktB* gene. Two additional PHA synthases encoded by the *phaC1* (BxeB0358) and *phaC2* (BxeC0053) genes are arranged in the *phaC2J1, phaC3J2* gene clusters and are phylogenetically distant from PhaC1 (Urtuvia et al. 2018). PhaCs are highly redundant in *Paraburkholderia* species, and their distribution among the Burkholderiales order shows four major groups (Álvarez-Santullano et al. 2021). Sequences of groups A and B are related to class I PHA synthases. Sequences of group C are related to class II PHA synthases. Sequences of group D are the most distant PhaCs from previously described classes. The PHA synthases from group D are present in several Burkholderiales species, encoded in a genomic context related to acetate metabolism, amino acid metabolic regulation, and fatty acid metabolism (Álvarez-Santullano et al. 2021). In *C. necator* H16, the PhaC2 (group D) participates in the synthesis of P(3HB) during low-oxygen stress. It is regulated by a universal stress protein (UspA) that is encoded next to the *phaC2* gene (Tang et al. 2022). A *Janthinobacterium* strain from Antarctica, belonging to the Burkholderiales order, harbors a functional PhaC2 classified within phylogenetic group D, whose activity increases at suboptimal growth temperatures, indicating a potential role in cold adaptation (Tan et al. 2020). These metabolic pathways suggest that *P. xenovorans* possesses the genes to synthesize diverse PHA copolymers from a wide range of carbon sources, which is part of the strategy to overcome stress upon nutritional imbalance.

Additionally, *P. xenovorans* LB400 harbors four intracellular PHA depolymerases (BxeA3308, BxeB2941, BxeB2846, and BxeA3270), which are similar to the depolymerases PhaZ1, PhaZ2, PhaZ6, and PhaZ7 described in *C. necator* H16 (Table S2). The PhaZ1 performs a thiolytic degradation of P(3HB) into crotonyl-CoA via (*R*)-3HB-CoA formation. Crotonyl-CoA may enter the *β*-oxidation of fatty acids via (*S*)-3-hydroxybutyryl-CoA, yielding acetyl-CoA and NADH (Eggers and Steinbüchel 2013). The PhaZ2, PhaZ6, and PhaZ7 depolymerases degrade P(3HB) into (*R*)-3-hydroxybutyrate ((*R*)-3-HB) monomers and P(3HB) oligomers (Abe et al. 2005, Gebauer and Jendrossek 2006, Sznajder and Jendrossek 2014). (*R*)-3HB is an important antioxidant molecule that protects cells against protein aggregation and cellular damage caused by ROS (Müller-Santos et al. 2021). (*R*)-3-HB can be reduced into acetoacetate by a (*R*)-3-HB dehydrogenase (HBDH). Acetoacetate can be activated into acetoacetyl-CoA, an intermediate of the *β*-oxidation of fatty acids and P(3HB) synthesis (Fig. 3). Finally, *P. xenovorans* harbors two oligomer hydrolases, PhaY1 and PhaY2, encoded by BxeA2223 and BxeA1368 that may degrade P(3HB) oligomers into (*R*)-3HB (Saegusa et al. 2002).

## Osmoadaptation to saline stress

During bioremediation, bacteria are subjected to challenges such as oxidative stress, nutrient scarcity, and high salinity, which increase cell osmolarity and reduce water activity, impairing microbial function and community stability (Stevenson et al. 2015, Kumar et al. 2022). Some bacteria develop adaptive responses to salinity, which may affect their pollutant degradation efficiency (Atoufi et al. 2020, Xu et al. 2023). Adaptation strategies include the maintenance of internal ion balance via transporters and pumps, and the relieve of membrane tension through mechanosensitive channels (Sweet et al. 2021, Wang and Blount 2023). Haloadaptation also involves transport systems such as ABC transporters, PutP, threonine efflux proteins, and ammonium transporters (Kaur and Kaur 2024). Salt stress may trigger the antioxidant response in bacteria (Smirnova et al. 2000), a well-known phenomenon in plants (Chourasia et al. 2021). Notably, some bacteria such as *Acinetobacter halotolerans, Halomonas cupida* J9, *R. erythropolis* sp. YHLT-2, *Bacillus* sp. Z-13, and *Bacillus licheniformis* degrade POPs under saline conditions (Longang et al. 2016, Zhang et al. 2023, Srimathi et al. 2024).

The second strategy in response to saline conditions involves maintaining a low intracellular salt concentration and balancing osmotic pressure by producing compatible solutes, such as betaine and ectoine (Schwibbert et al. 2011, Guo et al. 2019). Different Gammaproteobacteria strains have the metabolic capability to synthesize compatible solutes such as glycine betaine. In *Sinorhizobium meliloti* and *Halomonas elongata*, glycine betaine is synthesized from choline through a pathway encoded by the *betICBA* operon that involves the conversion of choline to betaine aldehyde and then to glycine betaine (Osterås et al. 1998, Cánovas et al. 2000). Representatives of the genus *Arthrobacter* possess the metabolic capacity to degrade PCBs, triazines, and benzene under fluctuating osmotic pressure conditions. As an adaptation strategy, *Arthrobacter* sp. B6 has an ABC-type glycine betaine/carnitine/choline and proline/betaine alkali transporters, enabling the accumulation of compatible solutes, including choline, glycine betaine, and valine, thereby increasing tolerance to osmotic and saline stress (Xu et al. 2017, Guo et al. 2019).

In addition, in saline environments, the cytoplasmic membrane of bacteria may significantly increase the acidic phospholipid cardiolipin content (Zhang and Rock 2008). Cardiolipin confers protection against saline stress and also against organic compounds (Zhang and Rock 2008, Dercová et al. 2019).

**Figure 4.**
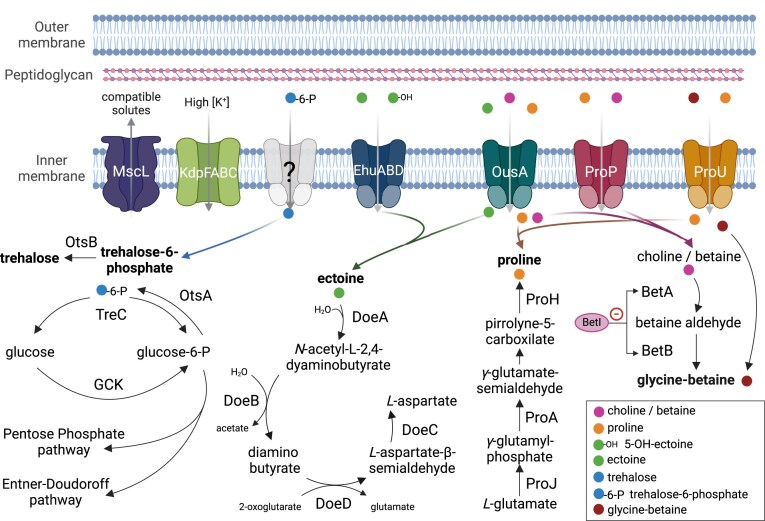
Prediction of adaptive responses to saline stress in *P. xenovorans* LB400. Under high osmolarity conditions, compatible solutes accumulate at high concentrations within the cell. After controlling turgor imbalance, large conductance mechanosensitive channels (MscL) rapidly release edible solutes and larger molecular metabolites. The ATP-dependent K^+^ transporter (KdpFABC) actively drives potassium into the cell under low intracellular K^+^ concentration conditions, contributing to the regulation of cellular osmotic pressure. The ectoine/5-hydroxyectoine transporter (EhuABD) allows the uptake of these compatible solutes, which can be metabolized in the *P. xenovorans* cell as a carbon and energy source through the expression of the *doeABDC* genes, yielding l-aspartate as the final product, and acetate, 2-oxoglutarate, and glutamate as metabolic intermediates. The transporters for compatible solute precursors (ProU, ProP, and OusA) enter glycine–betaine and l-proline and their precursors, such as choline/betaine, into the cell, which are dehydrogenated by the enzymes BetA and BetB to form glycine-betaine. BetI is the negative transcriptional regulator of the *betAB* genes, activated by increased glycine–betaine osmoprotectant concentration. ProU directly imports the osmoprotectant. Trehalose plays a dual role in cellular regulation under saline stress, acting either as a compatible solute (trehalose) in response to osmotic pressure fluctuations or as a carbon and energy source when it is metabolized into glucose and glucose-6-phosphate. The identified determinants associated with compatible solutes in the genome of *P. xenovorans* are highlighted in bold. Protein sequences obtained from the NCBI (National Center for Biotechnology Information) platform were selected and cross-referenced with the Uniprot KB–Swiss Prot database to determine molecular determinants associated with salinity using experimentally validated sequences as a filter. The cutoff values for positive alignments were set at identity and coverage higher than 40% and 70%, respectively. Created in https://BioRender.com.

Bacterial synthesis of compatible solutes prevents dehydration by restoring cell volume and turgor pressure in response to the increased salinity of the environmen. In this study, identification of molecular determinants in *P. xenovorans* associated with salinity was performed using the BLASTp tool (Altschul et al. 1990). Protein sequences obtained from the NCBI platform and compared with the Uniprot KB–Swiss Prot database, using experimentally corroborated sequences. The cutoff values for positive matches were >40% identity and >70% coverage. *Paraburkholderia xenovorans* possesses genes encoding proteins involved in the synthesis and transport of compatible solutes, such as glycine–betaine, proline, and trehalose (Fig. 4; Tables S3–S5). Through choline, proline/betaine transporters BetT, OusA, ProP, ProV, and ProW, choline, proline, and betaine molecules enter the cell (Kappes et al. 1996). The genes encoding ProP (BxeA1509), ProV (BxeB1616), and ProW (BxeB1615) were identified in *P. xenovorans* (Table S4). In contrast, the *betB* gene (glycine/betaine/choline transporter) is absent in the LB400 genome. The pathway associated with the synthesis of the compatible solute glycine/betaine, involving *betA* (choline dehydrogenase; BxeB1592), *betB* (betaine aldehyde dehydrogenase), and its regulator *betI* (BxeB1590), were identified, showing >95% identity to their homologs in *P. aromaticivorans* (Tables S3 and S5). In the context of PCB degradation, the metabolic reconstruction of glycin/betaine synthesis in *P. xenovorans* strain LB400 suggests that this metabolism may be associated not only with an adaptive response to saline stress but also as a strategy to alleviate oxidative stress generated by the combination of (i) PCB metabolism, and (ii) environmental stresses related to fluctuations in salinity, desiccation, and/or turgor pressure, similar to what may occur inside *P. aromaticivorans* cells during the degradation of aromatic and aliphatic hydrocarbons in saline environments (Lee and Jeon 2018, Lee et al. 2019). In *E. coli*, glycine–betaine and choline are the primary compatible solutes under hypersaline conditions (Sleator and Hill 2002).

*Paraburkholderia xenovorans* possesses genes associated with the trehalose synthesis pathway from glucose-6-phosphate, including OtsA (trehalose-6-phosphate synthase) and OtsB (trehalose-6-phosphate phosphatase) (Lee et al. 2023). Trehalose is one of the most prominent protein stabilizers in bacteria, protecting the native conformation of cytoplasmic proteins (Ruhal et al. 2013). Additionally, *P. xenovorans* has genes for the degradation of compatible solutes, which can be utilized as a carbon source through the PP and the ED pathways. This is another example of how *P. xenovorans* may modulate metabolism as an adaptive strategy to challenging environmental conditions (Fig. 4). Genes encoding ProH (pyrroline-5-carboxylate synthase), ProJ (γ-glutamate kinase), and ProA (γ-glutamyl phosphate reductase) were identified in the genome of *P. xenovorans*, which allow the aerobic synthesis of proline from glutamate (Pérez-Arellano et al. 2010).

In the genome of *P. xenovorans*, proteins encoded by the genes BxeC0063, BxeC0058, BxeC0060, and BxeC0061 have high identity (>55%) with the ectoine-degrading enzymes DoeA, DoeB, DoeC, and DoeD, however, genes encoding for ectoine synthesis were not identified. In *P. xenovorans*, the *doe* genes are not clustered in an operon (Table S3). The expression of the *doe* genes is associated with the use of ectoine as a carbon and energy source (Galisteo et al. 2024). The *doeA* and *doeB* genes are predominant in rhizobial Alphaproteobacteria and Burkholderiales.

The genomic search of saline stress-associated transporters identified proline/betaine transporter ProP, ABC proline/glycine transporter subunit ProV and inner membrane subunit ProW, and large-conductance mechanosensitive channel K^+^ transporters KdpFABC and MscL. An ectoine ABC transporter was identified with a high identity to its homolog in *P. susongensis* (Fig. 4; Table S4). In *E. faecalis* V583, in response to high salinity conditions, K^+^ is uptaken by the potassium-transporting ATPase (*kdpFABC* operon), which is regulated by the sensor kinase KdpD and transcriptional factor KdpE (*kdpED* operon), whereas Na^+^ is extruded by the V-type sodium ATPase (*ntpFIKECGABDJ* operon) (Acciarri et al. 2023).

In the context of bioremediation of saline soils contaminated with aromatic compounds, including PCBs, the metabolism of aromatic compounds by *P. xenovorans* LB400 and *P. aromaticivorans* BN5 may be linked to the osmoadaptation, including the biosynthesis of glycine/betaine. Osmoadaptation and glycine/betaine may play a role under increased salinity in (i) maintaining cellular osmotic balance (turgor pressure), (ii) stabilizing the cell membrane, and (iii) enhancing the antioxidant response (Figueroa-Soto and Valenzuela-Soto 2018). This suggests that the key functions of compatible solutes, such as glycine/betaine, extend beyond their well-established roles in salt and osmotic stress response. These strategies not only promote osmoprotection but also confer increased metabolic efficiency, improving the utilization of contaminants as carbon and energy sources under dynamic environmental stress conditions.

While most studies on salt stress responses in the genus *Paraburkholderia* focus on rhizobacteria, the findings presented in this review, involving species capable of degrading contaminants in complex, multistress environments, pave the way for understanding how hostile environmental conditions drive the evolution of conserved cellular defense mechanisms. Functional studies are required to characterize the oxidative and osmotic stress responses, the accumulation of compatible solutes, and the regulation of carbon and energy metabolism, which may together enhance survival and biodegradation efficiency in extreme environments.

## Synthesis of siderophores under iron limitation

Nutrient limitations, such as iron scarcity, are a source of exogenous stress, significantly impacting bacterial biodegradation by restricting growth and metabolic processes. Iron limitation is a challenge for bacterial growth and biodegradation, but also drives the evolution of adaptive strategies of microbial communities in nutrient-limited environments. Ferric ion, most commonly found in its Fe(III) oxidation state, has a low bioavailability due to its poor solubility at neutral pH in the presence of oxygen (Neilands 1995).

One of the most well-known strategies to support iron limitation is the production of siderophore compounds that have a high affinity and selectivity for Fe(III) (Hider and Kong 2010). Alternative mechanisms developed by prokaryotic strains include the synthesis of alternative electron transport chain components (Shafiie et al. 2022), Fur- and sRNA-based transcriptional regulation (Nelson et al. 2019), and cross-regulation with other nutrients (e.g. phosphonate, starch) (Cheng et al. 2021). Siderophores are low molecular mass (<2 kDa) Fe(III) carriers containing generally hydroxamate, α-hydroxycarboxylate, and catechol ligands to bind iron with high affinity under iron-limiting conditions (Neilands 1995).

Iron is an essential micronutrient for diverse metabolic processes such as respiration, the Krebs cycle, oxygen transport, DNA synthesis, nitrogen fixation, methanogenesis, and photosynthesis. Iron is part of the iron–sulfur clusters, di-iron centers, and heme cofactors of proteins. Under iron limitation, bacteria modify metabolic pathways, reducing the synthesis of iron-containing proteins and affecting overall metabolic efficiency, which can interfere with biodegradation processes (Dinkla et al. 2001, Mendonca et al. 2020, Yue et al. 2023).

In response to nutrient limitation, bacteria modify their metabolism, for example, by changing substrate preference. In iron-deficient soils, soil *Pseudomonas* species prioritize siderophore biosynthesis through a hierarchical carbon metabolism strategy, enhancing iron uptake from soil (Mendonca et al. 2020). This metabolic reprogramming involves increasing flux toward gluconeogenic substrates, which leads to a significant increase in siderophore synthesis and iron scavenging and a decrease in other metabolic processes (Mendonca et al. 2020). The activity of toluene monooxygenase and the lower pathway enzymes in the *P. putida* strains mt2 and WCS358 is significantly reduced under iron limitation, affecting toluene biodegradation efficiency (Dinkla et al. 2001).

Under iron-limiting conditions, *P. xenovorans* synthesizes a hydroxamate-type siderophore. The *mba* gene cluster from *P. xenovorans* strain LB400 encodes nonribosomal peptide synthetase (NRPS) and several transport genes for the siderophore (Vargas-Straube et al. 2016). The proposed structure of the malleobactin siderophore is l-Nδ-hydroxy-Nδ-formylOrn-d-β-hydroxyAsp-l-Ser-l-Nδ-hydroxy-Nδ-formylOrn-1,4-diaminobutane, which is closely related to malleobactin-type siderophores reported in *Burkholderia thailandensis. Paraburkholderia xenovorans* possesses the complete pathways to synthesize each of these components from central metabolism intermediates (e.g. pyruvate, acetyl-CoA, 2-ketoglutarate, and glycerate-3-phosphate) (Fig. 3B). The promoters in the *mba* gene cluster strongly suggest regulation by the ferric uptake regulator (Fur) protein and the alternative extracytoplasmic function sigma factor MbaF.

In *Burkholderia sensu lato*, diverse NRPS systems for siderophore synthesis have been described (Donadio et al. 2007, Thomas 2007). Malleobactin is produced by *Burkholderia pseudomallei, Burkholderia mallei*, and *B. thailandensis* (Alice et al. 2006, Franke et al. 2013, Franke et al. 2015). *Burkholderia vietnamiensis, Burkholderia cepacia, Burkholderia ambifaria*, and *Burkholderia cenocepacia* synthesized ornibactin (Meyer et al. 1995, Agnoli et al. 2006). Additional siderophores have been reported, such as cepabactin in *B. cepacia* (Meyer et al. 1995), pyochelin in *B. pseudomallei* (Alice et al. 2006), and cepaciachelin in *B. ambifaria* (Thomas 2007).

## Proteostasis network in *P. xenovorans* and degrading bacteria

**Figure 5.**
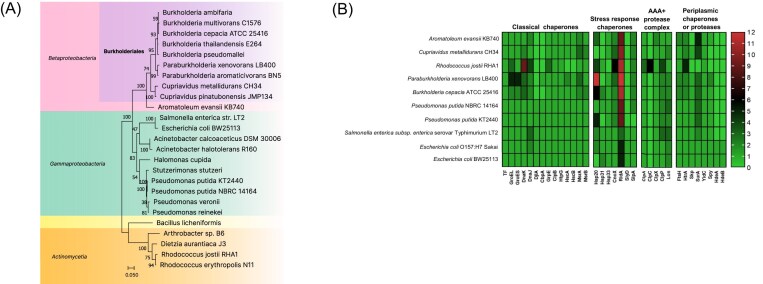
Evolutionary relationships of the bacterium *P. xenovorans* with other relevant aromatic-degrading bacterial strains, and abundance of genes encoding chaperones and proteases in their genomes. (A) Phylogenetic tree of aromatic-degrading Burkholderiales and other bacteria. Evolutionary phylogenetic tree of bacterial 16S rRNA genes were constructed with MUSCLE alignment (Edgar 2004) and Maximum-likelihood clustering (1,000 bootstrap). (B) The genomes of *P. xenovorans* LB400, *C. metallidurans* CH34, *B. cepacia* ATCC 25416, *P. putida* (NBRC 1416 and KT2440), *A. evansii* KB740, *R. jostii* RHA1, *S. enterica* serovar Typhimurium LT2, and two strains of *E. coli* (O157:H7 Sakai and BW25113) were evaluated. The heatmap indicates the number of gene copies. Genome sequences were obtained from the National Center for Biotechnology Information (NCBI) database. The strains and genome accession codes are listed in Table S1. Assessment of classical cytoplasmic chaperones (TF, DnaK, DnaJ, DjlA, CbpA GrpE, GroEL, GroES, HtpG, ClpB, HscAB, and MsrAB), membrane and periplasmic chaperones (HtrA, FstH, SurA, Skp, YidC, Spy, and HdeAB), stress response holdase chaperones (Hsp33, Hsp20, Hsp31, SlyD, SlpA, CnoX, and RidA), and proteolytic systems (ClpX/ClpA/ClpC/ClpP/ClpY/ClpQ, Lon) were performed by sequence comparative NCBI Blast tools (https://blast.ncbi.nlm.nih.gov/Blast.cgi). Conserved domains/motifs and ATP-binding sites were confirmed using CDART (Conserved Domain Architecture Retrieval Tool) and CDD (Conserved Domains) tools from NCBI portal (https://www.ncbi.nlm.nih.gov/cdd).

Proteostasis or protein homeostasis in bacteria refers to the highly flexible network involved in the biosynthesis, folding, trafficking, and degradation of proteins (Santra et al. 2017). Cellular proteostasis is supported by a highly flexible network that includes chaperones, proteases, and folding catalysts such as PPIases, oxidoreductases, and S–S bond isomerases. This network allows the modulation of the proteome to environmental changes toward an adaptive response (Voth and Jakob 2017). The proteostasis network is crucial for the adaptation to environmental stresses such as extreme temperatures and other harmful conditions (Ferrer et al. 2003). Chaperones play a key role in the cellular response against stress by supporting protein folding, preventing aggregation, and maintaining protein homeostasis (Hidalgo 1996, Goemans et al. 2018). Chaperones can be classified into two groups: (i) the foldase chaperones that participate in the folding of nascent proteins or unfolded mature proteins. With few exceptions, most of these chaperones are ATP-dependent and their functions ensure that many proteins attain their native conformation, and (ii) the ATP-independent holdase chaperones that act by binding exposed hydrophobic aminoacidic sequences of unfolded, partially folded, or misfolded proteins, therefore preventing their aggregation or degradation. The foldase group includes GroEL/ES (Ryabova et al. 2013), DnaK–DnaJ/CbpA/DjlA–GrpE complex (Genevaux et al. 2001, Azaharuddin et al. 2023), ClpB (Uchihashi et al. 2018), HtpG (Mangla et al. 2023), trigger factor (TF) (Wu et al. 2022), HscA/B (Puglisi and Pastore 2018), MsrA/B (Ezraty et al. 2005), and YidC (Zhu et al. 2013). The holdase chaperones group include Hsp20 (Sato et al. 2024), Hsp31 (Chatterjee et al. 2018), Hsp33 (Krewing et al. 2019), CnoX (Goemans et al. 2018), RidA (Müller et al. 2014), Skp and SurA (Thoma et al. 2015), Spy (Mitra et al. 2021), HdeA/B (Thapliyal and Mishra 2024), SlpA (Greitner et al. 2017), and SlyD (Kovermann et al. 2013). Some holdase chaperones are constantly active, while others are activated only under certain stress conditions (Kim et al. 2021). The gene copy number of chaperones such as heat shock protein 70 (Hsp70), Hsp60, Hsp40, and small HSP (sHsp) has increased during the evolution of each of the bacteria, archaea, and eukarya domains (Powers and Balch 2013). Modification of diverse chaperones through inserts and deletions (indels) (e.g. Hsp70 and Hsp60) has been tracked to the evolution of diverse bacterial taxa, which also indicates that Proteobacteria, including Burkholderiales, are more recently evolved bacterial taxa (Gupta 2016). Figure 5(A) presents a phylogenetic tree of aromatic-degrading and nondegrading bacterial strains, providing insights into their adaptive mechanisms to aromatic compounds, oxidative stress, and environmental stressful conditions within an evolutionary and taxonomic context.

Most of the unfolding and aggregation of proteins that occur during stress in bacteria are managed by upregulating the ATP-dependent chaperones. The most well-known chaperones include GroEL, DnaK, HtpG, and ClpB (Hartl et al. 2011). In *E. coli* cells, due to the inactivation of redox-regulated metabolic enzymes involved in ATP-generating pathways, the stress induced by ROS accumulation leads to a decrease of up to 50% in intracellular ATP concentration (Winter et al. 2005, 2008). A decrease in ATP levels impacts the activity of ATP-dependent chaperones and proteases. Consequently, the upregulation of an alternative proteostasis network, which includes ATP-independent chaperones, is a key strategy for managing oxidative stress.

The proteostasis network is shaped by foldase (mainly ATP-dependent) and holdase (ATP-independent) chaperones and proteases, which are grouped into four categories that include: classical chaperones, stress chaperones, AAA+ proteolytic complexes, and membrane/periplasmic chaperones or proteases. The proteostasis network was analyzed and compared in aromatic-degrading *P. xenovorans*, Burkholderiales strains, and other relevant bacteria in biodegradation and bioremediation (Fig. 5B; Table S1). For comparison, nondegrading pathogenic strains, such as *E. coli* strains O157:H7 and BW25113, and *Salmonella enterica*, were also analyzed. For this analysis, the complete genome of each bacterium was obtained from the National Center for Biotechnology Information (NCBI) database and bioinformatically inspected using SnapGene 6.0.2 software. Identified genes and predicted protein products were analyzed by sequence comparison using NCBI Blast tools (https://blast.ncbi.nlm.nih.gov/Blast.cgi). The sequence of every gene and protein was verified by alignment analysis using Clustal Omega (https://www.ebi.ac.uk/jdispatcher/msa/clustalo) and the COBALT Multiple Alignment Tool from NCBI (https://www.ncbi.nlm.nih.gov/tools/cobalt/cobalt.cgi). The presence of conserved domains/motifs and ATP-binding site of each identified protein was also confirmed using CDART (Conserved Domain Architecture Retrieval Tool) and CDD (Conserved Domains) tools from NCBI (https://www.ncbi.nlm.nih.gov/cdd). The main findings of this study are described below.

### Classical chaperones

The classical ATP-dependent foldase chaperone systems DnaK/DnaJ/GrpE and GroEL/GroES promote refolding of protein substrates (Castanié-Cornet et al. 2014). DnaK is involved in newly synthesized polypeptide folding, preventing protein misfolding and aggregation (Castanié-Cornet et al. 2014). DnaK binds to exposed hydrophobic regions of its substrates and promotes protein refolding in an ATP-dependent process regulated by the cochaperone DnaJ (Mayer and Bukau 2005) or its homologs CbpA or DjlA (Castanié-Cornet et al. 2014). As shown in Fig. 5, these genes are widely distributed and show high redundancy in model-degrading bacteria. Five copies of the DnaK encoding gene are present in the genome of *P. xenovorans*, and 10 copies are in the *R. jostii* genome, both remarkable PCB-degrading bacteria. In these strains, the *groES, groEL*, and *dnaJ* genes also exhibit higher redundancy compared with nondegrading bacteria, whereas the *dnaJ* gene homologs *cbpA* and *djlA* genes are absent in these degrading strains. Interestingly, DnaK, GroEL, and GroES are commonly induced during aromatic compounds degradation in *P. xenovorans* LB400 (Agulló et al. 2007, 2017, Martínez et al. 2007, Méndez 2017, Rodríguez-Castro et al. 2024). DnaK is a redox-sensitive chaperone with a crucial protective role during oxidative stress (Winter et al. 2005).

GrpE is a nucleotide exchange factor in the DnaK/DnaJ system, playing an important role in regulating the activity of the DnaK/DnaJ system in response to thermal stress (Groemping and Reinstein 2001). The *grpE* gene is in a single copy in all the analyzed bacterial strains except for *R. jostii* (three copies). Together with the DnaK/DnaJ/GrpE system, ClpB is an ATP-dependent heat shock protein that plays an essential role in rescuing damaged proteins from large aggregates (Barnett et al. 2000, Lee et al. 2003). As shown in Fig. 5(B), a single copy of the *clpB* gene is present in all bacterial strains. ClpB is induced in *P. xenovorans* strain LB400 by the aromatic compound *p-*cymene (Agulló et al. 2017), which may have the function of assisting damaged proteins generated upon *p-*cymene exposure.

The GroEL and GroES chaperones promote folding of almost 250 proteins in *E. coli*, representing 10%–15% of its total cytosolic proteins (Kerner et al. 2005). As shown in Fig. 5(B), both *groEL* and *groES* genes are present in five copies in *P. xenovorans*. In contrast, in most genomes, these genes are in a single copy. Multiple gene copies of these key chaperones may be part of the adaptive strategy of *P. xenovorans* against different stressful conditions.

HtpG is a chaperone foldase that promotes the folding and activation of newly synthesized proteins, prevents aggregation, and facilitates disaggregation and refolding of misfolded and aggregated proteins. The HtpG chaperone is upregulated under oxidative stress and is involved in bacterial swarming, biofilm formation, cell division, and pathogenicity (Genest et al. 2019, Wickner et al. 2021). This study indicates that the *htpG* gene is present in all bacterial strains as a singleton (Fig. 5B). Interestingly, the HtpG chaperone is induced in *P. xenovorans* by 4-chlorobenzoate (Martínez et al. 2007), suggesting a relevant role in cell protection during degradation of aromatic compounds.

The redox-sensitive methionine sulfoxide reductases (Msr) are thioredoxin (Trx)-dependent oxidoreductase enzymes that repair oxidized proteins at methionine (Met-O) residues, participating in the refolding and recovery of proteins damaged during oxidative stress (Boschi-Muller 2018). Up to three copies of the *msr* genes are present in *P. xenovorans* and other degrading bacteria (Fig. 5B).

Finally, the TF is the only bacterial chaperone that binds to ribosomes. TF is transiently associated with ribosomes in a 1:1 stoichiometry, binding to and acting on nascent polypeptides emerging from the ribosome (Rutkowska et al. 2008). Approximately 70% of proteins fold to their native structures after association with the TF. Hence, its copy number is expected to be conserved in all bacterial genomes (Fig. 5B).

In summary, classical chaperones are widely distributed in bacteria and shown to be one of the strategies most used by *P. xenovorans* and degrading bacteria to cope with the stress generated by the presence of aromatic compounds. A higher redundancy of genes encoding GroEL, GroES, DnaK, DnaJ, and MsrA chaperones is present in the model-degrading bacteria *P. xenovorans* and *R. jostii*, compared with enteric bacteria (nondegrading). Accordingly, these are also the most upregulated chaperones during aromatic metabolism in the model-degrading bacteria *P. xenovorans* and *P. putida* (Segura et al. 2005, Denef et al. 2006, Domínguez-Cuevas et al. 2006, Martínez et al. 2007, Agulló et al. 2017).

## Stress response chaperones

### Holdase chaperones

Environmental stress can lead to the accumulation of ROS, a decrease in ATP intracellular levels, and the enzymatic activity of ATP-dependent chaperones. Therefore, ATP-independent holdase chaperones are induced as a strategy to cope with these stressful conditions and prevent protein aggregation (Hoffmann et al. 2004, Thoma et al. 2015, Goemans et al. 2018). These molecular chaperones are regulated at the transcriptional and/or posttranslational level under stress, which allows them to respond rapidly and protect the integrity of the bacterial proteome (Voth and Jakob 2017). Holdase chaperones form a stable complex with damaged proteins, preventing their irreversible aggregation while the stress persists. Once the unfavorable condition subsides, the chaperones return to their inactive state and release the bound protein, which then folds itself. Although holdase chaperones lack refolding activity, this mechanism provides a means to prevent the accumulation of misfolded proteins and to protect the cells against the toxicity associated with protein misfolding. Chaperones that are generally involved in stress responses include the heat shock proteins RidA, SlyD, SplA, CnoX, and Hsp. RidA is part of a reversible redox mechanism, possessing a regular function in the absence of stress. Under nonstress conditions, RidA functions as a deaminase by releasing ammonia from reactive enamine/imine intermediates (Lambrecht et al. 2012). In the presence of reactive chlorine species, RidA chlorinates positively charged amino acids, preventing aggregation of misfolding substrates (Voth and Jakob 2017). The *ridA* gene is highly redundant in most strains, reaching up to 12 copies in *P. xenovorans* LB400 (Fig. 5B), which may have a key role during oxidative stress generated by the catabolism of PCBs and chlorinated aromatic compounds. A higher redundancy of *ridA* is evident in model-degrading bacteria (9–12 copies) compared to nondegrading enteric bacteria (3–9 copies), suggesting the physiological relevance of RidA on protein homeostasis under stress related to biodegradation.

Another chaperone widely distributed among bacteria is SlyD, a member of the peptidyl-prolyl isomerase (PPIase) family (Quistgaard et al. 2016). SlyD is structurally composed of the PPIase domain that catalyzes peptidyl-prolyl *cis-trans* isomerization, accelerating the slow steps in protein folding, while the C-terminal domain performs chaperone activity (IF; insert-in-flap), which prevents aggregation of cytosolic proteins (Kim et al. 2010). Interestingly, the *slyD* gene is present only in enteric bacteria but absent in all analyzed degrading bacteria (Fig. 5B). SlpA, the SlyD-like protein A, is an FKBP-type peptidyl-prolyl *cis-trans* isomerase, but unlike SlyD, it lacks the C-terminal metal-binding region (Hottenrott et al. 1997, Löw et al. 2010). SlpA possesses moderate PPIase activity, significantly weaker than SlyD (Hottenrott et al. 1997, Geitner et al. 2017). In contrast to SlyD, the chaperone SlpA is highly stable under hostile cellular conditions (Geitner et al. 2017), which is relevant for proteostasis under stress. Two *slpA* gene copies are present in *P. xenovorans* and all model-degrading bacteria, except for *R. jostii* (no copies) and enteric bacteria (one copy). A PpiB protein with PPIase activity has been described in *B. pseudomallei* (Bzdyl et al. 2019). Deletion of *ppiB* leads to pleiotropic effects, including increased sensitivity toward multiple antibiotics and the loss of several virulence determinants (Bzdyl et al. 2019), revealing its essential role in proteome homeostasis and virulence.

During oxidative stress, cysteine ​​and methionine residues of proteins can be oxidized, leading to protein inactivation or misfolding. Therefore, oxidoreductases also contribute to proteostasis by rescuing redox-sensitive residues from oxidation (Dahl et al. 2015). CnoX (YbbN) oxidoreductase is a multidomain protein with a dual function that prevents irreversible protein aggregation and protects cellular proteins from hyperoxidation. Upon stress, it transfers its substrates to DnaK/DnaJ/E and GroEL/GroES for refolding (Meireles et al. 2024). CnoX is the only known holdase directly cooperating with the essential GroEL/GroES complex and is involved in the response to oxidative stress induced by hypochlorous acid (HOCl) (Goemans et al. 2018), hydrogen peroxide (Muñoz-Villagrán et al. 2024), and heat stress (Izquierdo-Fiallo et al. 2023). Interestingly, the Burkholderiales strains *P. xenovorans* and *C. metallidurans* harbor three copies of the *cnoX* gene, whereas nondegrading bacteria have only one copy (Fig. 5B). The aerobic metabolism of aromatic compounds generates ROS that may induce oxidative stress. The presence of multiple copies of the chaperedoxin CnoX-encoding gene could contribute to achieving an increased intracellular concentration of CnoX, thereby enhancing the protection of its target proteins or increasing the number of substrate proteins to be protected by this chaperedoxin when bacteria are in contact with aromatic compounds.

### Small heat shock proteins

The small heat shock proteins (sHsps) are low-molecular-weight holdase chaperones, ranging from 12 to 43 kDa, initially described as heat shock proteins (Jacob et al. 2017). They protect against stressful environmental conditions in prokaryotic and eukaryotic cells (Maleki et al. 2016, Jacob et al. 2017). Most studied sHsps in prokaryotes include Hsp20, Hsp31, Hsp33, and Spy (Kumsta and Jakob 2009, Singh et al. 2014, Aslam and Hazbun 2016, He et al. 2021).

The chaperones Hsp33 and Hsp31 play a major role in the oxidative stress response in *E. coli*, preventing protein aggregation (Voth and Jakob 2017). In *E. coli*, Hsp33 is activated during oxidative stress through the formation of two intramolecular disulfide bonds, the release of the bound zinc, and conformational rearrangements (Reichmann et al. 2018). In the absence of stress, Hsp33 is compactly folded, four cysteines of the C-terminal are reduced, and zinc is bound (Reichmann et al. 2018). Hsp31 is also involved in acid stress (Mujacic et al. 2004). The *hsp33* and *hsp31* genes are present as a single copy in the genomes of *P. xenovorans* and most of the model-degrading bacteria (Fig. 5B).

The Hsp20 chaperone, known as IbpA and IbpB in *E. coli*, is constitutively active and forms stable complexes with its substrates under stress conditions. *Escherichia coli* IbpA and IbpB cooperate to stabilize intermediate states of denatured proteins, thus promoting the efficiency of the disaggregating mechanisms DnaK/DnaJ/GrpE and ClpB (Ratajczak et al. 2009) and helping to maintain the activity of several enzymes during stress conditions induced by ROS, heat, and freeze–thaw (Kitagawa et al. 2002). Hsp20 suppresses protein aggregation at elevated temperatures in *Deinococcus radiodurans* (Bepperling et al. 2012). In *E. coli*, the expression of *hsp20* is controlled by RpoS or RpoH, which are master regulators of general stress responses and heat shock, respectively (Tilly et al. 1986, Cocotl-Yañez et al. 2014). In *A. vinelandii*, Hsp20 is also involved in desiccation resistance (Cocotl-Yañez et al. 2014). Hsp20-encoding genes are widely distributed and highly redundant in model-degrading bacteria, including *P. xenovorans*, with a maximum of 12 copies (Fig. 5B). The high redundancy of *hsp*20 in degrading bacteria suggests that this holdase chaperone plays a pivotal role in protein protection and cell proteostasis under different stress conditions, including abiotic factors.

### Membrane/periplasmic chaperones or proteases

In bacteria, the proteostasis of the membrane proteins is carried out by periplasmic or membrane chaperones and proteases, such as HtrA, SurA, Skp, YidC, and FtsH. HtrA (also known as protease Do or DegP) is present in all three domains of life, usually encoded by multiple gene copies (Muley et al. 2019). This protein has a dual activity, acting as a protease and chaperone in the periplasm. However, since the ATP is not available in the periplasm, it is presumed that HtrA could act as an ATP-independent chaperone (Zarzecka et al. 2019). HtrA activity is essential for bacterial survival in stressful environments, contributing to tolerating harsh conditions such as increased oxidative stress, osmotic stress, high temperatures, or extreme pH (Zarzecka et al. 2019). According to the results of this study, the protease/chaperone HtrA is encoded in the genome of all the analyzed bacterial strains, showing two copies in *P. xenovorans* and a maximum of five copies in *R. jostii*. Multiple copies of *htrA* may guarantee the protection and functionality of proteins (Fig. 5B).

SurA is a holdase chaperone essential for cell survival; specialized in the transport of unfolded proteins from the inner to the outer membrane of Gram-negative bacteria, and also in the periplasm under stress conditions (Mas et al. 2019). SurA mediates the folding of proteins translocated to the periplasm, combining both peptidyl propyl isomerase and chaperone functions. A multiple copy number of *surA* is present in the genome of Burkholderiales strains (three and four copies in *P. xenovorans* and *C. metallidurans*, respectively), suggesting that SurA may play a crucial role in the protection of periplasmic proteins under different types of stress in degrading bacteria.

YidC is a transmembrane protein that is involved in the insertion of membrane proteins into the lipid bilayer in bacteria under stressful conditions, acting as a holdase chaperone by interaction with hydrophobic domains of damaged or unfolded proteins, therefore, its absence results in the accumulation of aggregated or misfolded proteins in the cytoplasm and the inner membrane (Zhu et al. 2013). YidC functions as a ribosome receptor that directly accepts membrane proteins for their subsequent insertion (Dalbey et al. 2014). Figure 5(B) shows that all bacterial genomes possess one copy of the *yidC* gene.

Finally, FtsH is a highly conserved zinc-dependent metalloprotease located in the inner membrane that belongs to the AAA+ type ATPase family. *Escherichia coli* FtsH is the best-studied of all known members and is the only protease essential for growth and survival in bacteria. FtsH is involved in the quality control of specific membrane proteins (Akiyama 2009) and plays an important role during protein aggregation under heat shock conditions (Langklotz et al. 2012). *Paraburkholderia xenovorans* possesses two copies of the *ftsH* genes (Fig. 5B).

The multiple gene copies present in *P. xenovorans* reflect a specific adaptation for survival to satisfy the requirements of folding, protection, and recycling of proteins in the periplasm and extracellular matrix.

### Proteolytic ATPase complexes (AAA+)

The AAA+ proteins are ATPases associated with various cellular activities that contain a conserved ATP-binding domain (Hanson and Whiteheart 2005). The AAA+-containing proteolytic complex includes the well-known ClpAP, ClpCP, and ClpXP proteases, as well as the Lon protease. Clp proteins comprise the catalytic and substrate recognition subunits. For example, the protease ClpAP consists of the catalytic ClpP and the recognition of ClpA subunits (Kim et al. 2022). These proteases are composed of rings of several subunits that form a cavity, where the target protein is degraded. The Clp complex recognizes specific hydrophobic regions of misfolded or unfolded proteins to prevent their aggregation in the cytoplasm. ClpA, ClpC, and ClpX recognition subunits can also work alone as ATP-independent chaperones, thus constituting key regulatory components of the serine protease ClpP since they can rescue and fold proteins or present them to catalytic subunits for further degradation (LaBreck et al. 2017). These systems have an important role in the degradation of proteins and the recycling of their components. Genes of the AAA+ Clp protease complexes are present in all bacterial strains except the *clpC* gene (Fig. 5). While the *clpP, clpX*, and *clpA* genes are widespread, the *clpC* gene is present only in the Actinomycetota *R. jostii*. This agrees with a previous report in which *clpC* was detected only in Bacillota and Actinomycetota phyla (Nishimura and van Wijk 2015). ClpX uses multivalent strategies to discriminate between substrates that are in their native conformations or that are unfolded (LaBreck et al. 2017). Therefore, when the correct folding of their substrate proteins is not achieved, a specific signal is recognized that directs them to the ClpP protease for degradation. In *E. coli*, ClpXP is associated with protein aggregates (LaBreck et al. 2017). The *clpP* gene is present in two copies in *P. xenovorans* and in one to four copies in other bacteria (Fig. 5B).

Lon (La) is an ATP-dependent protease that degrades abnormal proteins or proteins that are no longer necessary for the cell (Simmons et al. 2008). In *E. coli*, this protease is responsible for 70%–80% of proteolysis in the cytosol (Maurizi 1992) and is required to maintain homeostasis and cell survival under stressful conditions. In bacteria, Lon plays a role in processes like motility, DNA replication, sporulation, and pathogenicity (Fu et al. 1997, Izquierdo-Fiallo et al. 2023). Additionally, Lon protease activity has been reported to increase resistance to harsh conditions such as nutrient starvation, oxidative stress, bacteriophage lysogeny, thermal stress, osmotic stress, and radiation (Fu et al. 1997, Takaya et al. 2003, Xie et al. 2016, Figaj et al. 2020). As shown in Fig. 5(B), *P. xenovorans* and most strains contain from two to three copies; in contrast, *A. evansii* and *R. jostii* possess a single copy. These genes that encode proteases are widely distributed, and there is a redundancy of ClpP and Lon that may be indicative of the importance of proteolytic degradation in the turnover of proteins.

**Figure 6.**
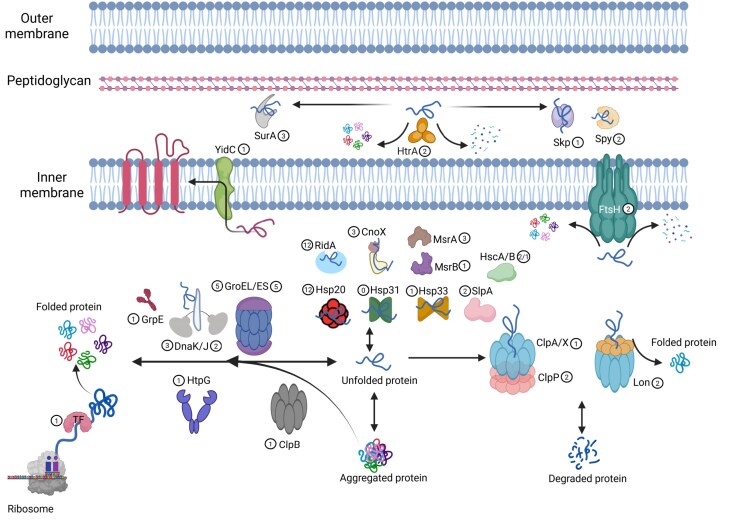
Schematic representation of the predicted proteostasis network in *P. xenovorans*. Newly synthesized proteins emerge from the ribosome and are assisted by trigger factor (TF) and chaperone systems such as DnaK/J, GrpE, and GroEL/ES for proper folding. Unfolded proteins are managed by a network of chaperones and proteases that mediate refolding or degradation. Numbers next to each factor correspond to the number of copies identified in the genome of *P. xenovorans*. Created in https://BioRender.com.

Overall, a remarkable feature of degrading bacteria is the redundancy of genes for specific chaperone systems, especially the classical and stress response chaperones. Particularly *P. xenovorans* has a high redundancy (>3 copies) of the *groEL, groES, dnaK, hsp20, ridA, msrA, surA*, and *cnoX* genes, which may represent an adaptive strategy to protect proteins from aggregation under oxidative stress conditions. The *msrA* gene was found in three copies in the Burkholderiales strains. The 12 copies of *hsp20* and *ridA* genes found in *P. xenovorans* were nonidentical and present in different genetic contexts, suggesting their functional differentiation that could significantly contribute to a higher flexibility of responses to different environmental challenges. In this context, we propose that holdases Hsp20 and RidA may be relevant to coping with stress. Moreover, the presence of different protein variants, such as those detected for MsrA and ClpPX, may confer additional adaptive responses through the proteostasis network. The model for the proteostasis network in *P. xenovorans* of the Burkholderiales order is shown in Fig. 6 . The interaction of molecular chaperones with unfolded and aggregated proteins generated during stress within the cell and the inner membrane is indicated, along with the gene copy number in *P. xenovorans*. These findings provide novel insights into the diversity and abundance of genes related to the proteostasis network in *P. xenovorans* LB400 and other members of the Burkholderiales order and offer new perspectives about the functionality and relevance of these chaperone systems to thrive in challenging environments. The multiple gene copies observed may be paralogous due to duplication events occurring before or after speciation (Chain et al. 2006). In addition, horizontal transfer of orthologous genes and genomic islands has been described in *P. xenovorans* LB400 and other members of the Burkholderiales order (Chain et al. 2006, Pérez-Pantoja et al. 2012). This leads to a variety of evolutionary scenarios that have ultimately shaped the multicopy patterns observed. The underlying mechanisms that led to this multicopy scenario in degrading bacteria require further investigation.

**Figure 7.**
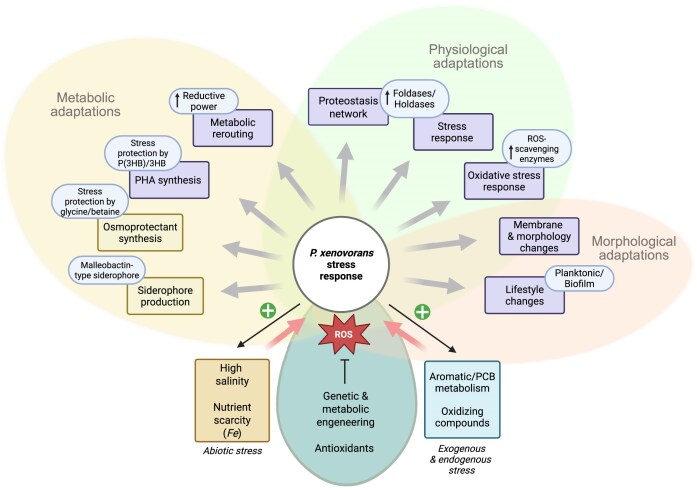
Comprehensive stress response mechanisms of *P. xenovorans* to aromatic compounds and abiotic stressors. The diagram illustrates the metabolic, physiological, and morphological adaptations of *P. xenovorans* to chemicals (aromatic compounds, PCBs, and oxidizing compounds) and abiotic stressors (high salinity and nutrient scarcity). Adaptive metabolic responses include the increase of reductive power, synthesis/degradation of P(3HB), synthesis of siderophores. Adaptive physiological responses include the upregulation of foldases and holdases, and ROS-scavenging enzymes. Adaptive morphological responses include membrane and cell morphology changes, along with lifestyle changes (planktonic and biofilm). Genetic and metabolic engineering approaches highlight potential enhancements of bacterial stress tolerance, wherein antioxidants play a role in mitigating oxidative stress. Overcoming stress strategies play a key role in enhancing the biodegradation of pollutants and bacterial tolerance to abiotic stress. Created in https://BioRender.com.

## Membrane, morphology, and lifestyle adaptations to aromatic compounds

Aromatic compounds cause changes in the membrane of bacterial cells, affecting their viability and fitness (Sikkema et al. 1995, Schweigert et al. 2001, Cámara et al. 2004). The toxic effect produced in bacteria by aromatic compounds and hydrocarbons is related to their accumulation in the cytoplasmic membrane (Heipieper and Martínez 2010). Solvents with a logP between 1 and 4, such as monocyclic aromatic compounds and phenols, are generally toxic to cells (Cámara et al. 2004, Heipieper and Martínez 2010). Compounds with extremely low solubility (e.g. high molecular PAHs) are not bioavailable enough to produce toxic effects on the membranes (Weber et al. 1994, Cámara et al. 2004, Heipieper and Martínez 2010). Due to their lipophilic nature, aromatic compounds tend to arrange between the phospholipid chains, producing an expansion of the cytoplasmic membrane that leads to a nonselective permeability to ions. The protons in their gradient dissipate, reducing their proton driving force and inhibiting the respiratory enzymes (Sikkema et al. 1992, Weber and de Bont 1996).

*Paraburkholderia xenovorans* LB400 incubation with the aromatic compounds 4-chlorobiphenyl, biphenyl, *p*-cymene, and *p*-cumate induces fuzzy outer and inner membranes and an increased periplasm (Agulló et al. 2007, 2017). Dihydroxybiphenyl is highly toxic for *P. xenovorans* and other bacteria, drastically reducing their viability (Cámara et al. 2004). The main target of PCBs and their dihydroxylated metabolites is the cytoplasmic membrane (Sikkema et al. 1995, Cámara et al. 2004). Dihydroxylated aromatic metabolites are hydrophobic and weakly acidic and may be uncouplers by a protonophoric shuttle mechanism (Schweigert et al. 2001). Cell membrane damage in bacteria is evidenced by inhibition of growth and an increased accumulation of lipids. However, the addition of the antioxidant α-tocopherol improves cell integrity and membranes upon exposure to 4-chlorobiphenyl (Ponce et al. 2011). This suggests that α-tocopherol may be exerting a protective effect on maintaining cell integrity.

The homeoviscous adaptation is a mechanism that modifies the permeability of the membrane to optimize growth and minimize energy expenditure (de Mendoza and Pilon 2019). Bacteria decrease the fluidity of the cytoplasmic membrane upon exposure to high concentrations of toxic organic compounds such as aromatic molecules (Heipieper and de Bont 1994, Cámara et al. 2004). The activation of the oxidative stress response along with fatty acid metabolism and changes in membrane lipid composition have been associated with the catabolism of aromatic compounds in bacteria (Denef et al. 2005, Navarro-Llorens et al. 2005, Mrozik et al. 2006, Patrauchan et al. 2008). Physiological adaptations protect bacteria during the metabolism of aromatic compounds.

Adaptation mechanisms of bacterial membranes to aromatic compounds and other toxic molecules include: (i) modification of the saturation of fatty acids in membrane phospholipids, (ii) fast *cis–trans* isomerization of unsaturated fatty acids, (iii) changes in the polar group of phospholipids, (iv) changes in cyclopropane and branched fatty acids, and (v) release of outer membrane vesicles from the cell surface (Eberlein et al. 2018, Dercová et al. 2019). However, in the *Paraburkholderia* genus, studies of membrane adaptation mechanisms to aromatic compounds are scarce.

### Modification of the saturation of fatty acids

The saturation of fatty acids in the membrane may be the main adaptive mechanism in bacteria exposed to aromatic compounds, compensating for the elevation of permeability induced by these molecules (Heipieper et al. 1992). In *Pseudomonas*, two fatty acyl desaturases (DesA and DesB) play a role in the unsaturation of fatty acids in the membrane, adding a *cis* double bond to fatty acids (Zhang and Rock 2008). DesA desaturates acyl-CoAs of membrane phospholipids, whereas DesB adds a double bond to acyl-CoAs derived from exogenous fatty acids. Fatty acyl desaturases are regulated by the presence of organic compounds, temperature, and other environmental conditions (Zhang and Rock 2008).

The whole-cell fatty acid profile of *P. xenovorans* LB400 is composed of 14:0 (4.7%), 14:0 3OH (8.5%), 16:1 7c (19.1%), 16:0 (18.2%), 17:0 cyclo (5.1%), 16:1 2OH (2.2%), 16:0 2OH (2.2%), 16:0 3OH (7.1%), 18:1 7c (27.3%), 18:0 (0.5%), 19:0 cyclo 8c (3.6%), and 18:1 2OH (0.9%) as main components (Goris et al. 2004). *Paraburkholderia xenovorans* LB400 cells decreased unsaturated fatty acid ratios, predominantly 18:1 and 16:1, when cells are grown on biphenyl or benzoate compared with succinate-grown cells (Parnell et al. 2006).

On the other hand, in *P. xenovorans* LB400 cells incubated with PCBs, the presence of biphenyl counteracts the effects of PCBs, elevating unsaturated fatty acids, with an increase in unsaturated phosphatidyl ethanolamine but a decrease in unsaturated phosphatidylcholine, which is a minor membrane component (Zorádová-Murínová et al. 2012). In contrast, membrane lipid saturation increases in the presence of carvone and terpenes of orange peel. The presence of biphenyl in a *Stutzerimonas stutzeri* culture incubated with PCBs increases membrane unsaturated fatty acids, including unsaturated phosphatidyl ethanolamine and phosphatidylcholine (Zorádová-Murínová et al. 2012). Incubation with phenol of *P. putida* P8 elevates the saturation degree of membrane fatty acids, decreasing the membrane permeability (Heipieper et al. 1992). Naphthalene increases the saturation degree of membrane fatty acids of *S. stutzeri* and *Pseudomonas* sp. JS150; in contrast, naphthalene decreases the saturation of membrane fatty acids in *P. veronii*. (Heipieper et al. 1992, Mrozik et al. 2006).

### Fatty acid *cis–trans* isomerization

In bacteria, a fast isomerization of *cis–* into *trans-*unsaturated fatty acids catalyzed by a *cis–trans* isomerase (Cti) leads to a rigidification of the membrane, decreasing the permeability of organic compounds that may intercalate and destabilize the membrane (Heipieper et al. 1992, 2003). Fatty acid *cis–trans* isomerization is an adaptation mechanism influenced by the concentration and hydrophobicity of the organic compounds, which occur *in situ* and do not require *de novo* synthesis of fatty acids (Eberlein et al. 2018, Dercová et al. 2019). The *trans-*unsaturated fatty acids cannot be converted into *cis-*unsaturated fatty acids by Cti; therefore, *de novo* biosynthesis of *cis-*unsaturated fatty acids is required (Zhang and Rock 2008).

In *P. xenovorans*, incubation with PCBs induces *trans* configuration of unsaturated fatty acids, whereas the addition of biphenyl, limonene, and the terpenes from orange peels to cells incubated with PCBs decreases *trans* unsaturated fatty acids (Zorádová-Murínová et al. 2012). *Pseudomonas putida* strains cultured in organic solvents, for example, toluene, increase the *trans* configuration of unsaturated fatty acids, while the cyclic and saturated fatty acids are not significantly modified (Dercová et al. 2019).

### Changes in the polar group composition

The composition of phospholipid polar head groups in the membrane is regulated by enzymes to balance the proportion of zwitterionic phospholipids (e.g. phosphatidylethanolamine, phosphatidylcholine, and glucosyldiacylglycerol) and acidic phospholipids (e.g. phosphatidylglycerol and cardiolipin) (Zhang and Rock 2008). These enzymes are intrinsic membrane proteins or proteins associated with the membrane. Cytosine diphosphate-diacylglycerol is a central intermediate in phospholipid synthesis that is formed by cytidine diphosphate diacylglycerol synthase (CdsA) from phosphatidic acid and cytosine triphosphate (Zhang and Rock 2008).

Aromatic solvents such as benzene and toluene reduce the transition temperature of phospholipids from a more ordered gel-lamellar phase to a less structured liquid–crystalline phase. To stabilize the fluidity in response to aromatic compounds, bacteria modify the membrane composition according to phospholipids with more suitable polar groups, favoring acidic phospholipids.

*Stutzerimonas stutzeri* and *P. veronii* cells grown on PCBs increase phosphatidylglycerol and phosphatidylcholine compared to glucose-grown cells, decreasing phosphatidylethanolamine (Murínová et al. 2014, Dercová et al. 2019). In *P. xenovorans* incubated with PCBs, the addition of biphenyl counteracts the effects of PCBs, increasing phosphatidylethanolamine and phosphatidylcholine and decreasing acidic phospholipids (Zorádová-Murínová et al. 2012). In *S. stutzeri* incubated with PCBs, the presence of natural terpenes from ivy leaves increases phosphatidylethanolamine and phosphatidylcholine (Zorádová-Murínová et al. 2012). Incubation of *P. putida* with phenol or toluene causes a decrease in the unsaturated/saturated ratio of membrane fatty acids, a *cis–trans* isomerization, and modifications in phospholipids, increasing phosphatidylglycerol and cardiolipin (diphosphatidylglycerol) and decreasing phosphatidylethanolamine (Weber et al. 1994, Weber and de Bont 1996). *Stutzerimonas stutzeri* and *P. veronii* cells grown on 3-chlorobenzoate compared to glucose-grown cells reduce phosphatidylethanolamine (Dercová et al. 2019).

### Changes in cyclopropane and branched fatty acids

Formation of cyclopropane fatty acids in Gram-negative bacteria have effects on transition temperature, comparable to the branching of fatty acids (*anteiso/iso* branching) in Gram-positive cells (Dercová et al. 2019). The cyclopropane group is formed through methylation of *cis*-unsaturated fatty acids by cyclopropane fatty acid synthase (Cfa), using *S*–adenosylmethionine as a methyl donor. Due to their steric properties, cyclopropyl fatty acids mimic *cis*-unsaturated fatty acids in the membrane, conferring an additional resistance mechanism to organic solvents and acid stress (Zhang and Rock 2008, Pini et al. 2009, Zorádová-Murínová et al. 2012).

*Paraburkholderia xenovorans* LB400 cells increased cyclopropyl fatty acids (C17 and C19) during incubation with biphenyl and benzoate compared with succinate-grown cells (Parnell et al. 2006). The degradation of PCBs also increased cyclopropyl fatty acids in *P. xenovorans* cells (Parnell et al. 2006). In *P. xenovorans* LB400 cells incubated with PCBs, the presence of biphenyl and limonene slightly increases the C17-cyclopropyl fatty acid content in the membrane, whereas the presence of biphenyl and ivy leaf terpenes increases the C19-cyclopropyl fatty acid content in membrane lipids (Zorádová-Murínová et al. 2012).

In *Pseudomonas* studies, a *P. putida* strain lacking the *cfaB* gene, encoding the synthesis of cyclopropane fatty acids, shows increased sensitivity to organic solvents, but not to other stressful conditions such as the presence of antibiotics, heavy metals, or temperature changes (Pini et al. 2009).

### Release of outer membrane vesicles

Release of outer membrane vesicles (OMVs) from the cell surface leads to a rapid increase in hydrophobicity, while cell aggregates and biofilm formation decrease the contact surface with toxic compounds (Eberlein et al. 2018). Vesicle formation in bacteria plays a key role against multiple stressors such as organic solvents (Baumgarten et al. 2012).

In *P. xenovorans* LB400, after incubation of succinate-grown cells with PCBs, vesicle formation was observed in a 3:4 ratio (vesicle/cell) (Parnell et al. 2006). *Burkholderia multivorans* C1576 in biofilms releases OMVs, which are enriched with lytic enzymes, siderophores, and antioxidant enzymes (Terán et al. 2020). The exposure of *P. putida* KT2440 to short- and long-chained *n*-alkanols revealed an up to 4-fold increase in OMV production (Eberlein et al. 2018).

### Lipopolysaccharide modifications

Due to the structural differences in the external cell membrane between Gram-negative and Gram-positive bacteria, different tolerances to solvents and toxic compounds have been observed (Harrop et al. 1989). Gram-negative bacteria showed a higher tolerance than most Gram-positive bacteria to a mixture of hydrocarbons, including saturated hydrocarbons (*n*-hexane, *n*-hexadecane, and cyclohexane), monoaromatic compounds (benzene, toluene, and ethylbenzene), and polyaromatic compounds (naphthalene, 2-methylnaphthalene, and fluorene) (Marilena-Lăzăroaie 2010). However, a specific taxon of Gram-positive bacteria called Mycolata (*Rhodococcus, Mycobacterium, Nocardia, Corynebacterium, Gordonia, Dietzia, Skermania*, and *Tsukamurella*) is extremely resistant to toxic hydrophobic compounds (Murínová and Dercová 2014, Méndez et al. 2022b). The cell wall has a unique composition, with a main component, arabinogalactan polysaccharide, which is linked with large 2-alkyl 3-hydroxy branched-chain fatty acids called mycolic acids. This complex is responsible for cell surface hydrophobicity and its very low permeability.

Gram-negative bacteria have lipopolysaccharides (LPS) as a main component of their external wall, constituted by a lipid fraction with saturated fatty acids and characteristic oligosaccharides and polysaccharides. Due to its high hydrophobicity, LPS of the cell wall confers Gram-negative bacteria a lower sensitivity to aromatic compounds such as PCBs, biphenyl, toluene, or benzene (Inoue et al. 1991, Weber and de Bont 1996, Murínová et al. 2014). The presence of the specific cations Mg^2+^ and Ca^2+^ promotes hexagonal phase formation by anionic phospholipids, increasing cell survival in the presence of toluene (Inoue et al. 1991, Weber and de Bont 1996). LPS-altered mutants or chemical loss of part of the LPS decreases the cellular protection to hydrophobic antibiotics and detergents in *E. coli* and *S. enterica* serovar Typhimurium (Hancock 1984, Harrop et al. 1989, Weber and de Bont 1996). However, the exclusion of the O-antigen from the molecule has no significant implication in tolerance to toluene, octanol, *p*-xylene, propylbenzene, and heptane compared to the wild strain (Junker et al. 2001, Ramos et al. 2002). In *P. putida* strain Idaho, a modification in the molecular pattern of the LPS on cells grown on *o*-xylene was observed, decreasing the molecular mass of LPS (Pinkart et al. 1996).

### Cell morphology modifications

The modification of cell shape and size is a mechanism of cellular resistance to the catabolism of aromatic compounds in Gram-negative and Gram-positive bacteria. A change in the elongated shape of bacillary bacteria to more coccoidal forms has been observed in *P. putida* P8 grown on phenol and 4-chlorophenol and *Rhodococcus* cells grown on *p*-cresol and *o*-cresol (Neumann et al. 2005, Gerginova et al. 2023). The coccoidal cells showed a decrease in the relative surface area of the cell in contact with the solvent, decreasing the toxic effects on its membrane. The scarcity of nutrients such as nitrogen, phosphorus, or water, as well as the presence of aromatic compounds as the only carbon and energy source, results in cell size modification, changing rod-shaped cells to coccal or filamentous forms (Seeger and Jerez 1993a, Gerginova et al. 2023). *Gordonia* sp. 12/5 grown on monophenols shows structural changes in cell populations, decreasing their average cell lengths compared to cells grown on glucose (Gerginova et al. 2023). In contrast, *Acidithiobacillus ferrooxidans* during phosphate starvation increases the size of cells 10-fold and induces diverse proteins, including an outer membrane porin (Seeger and Jerez 1993a, b). The same effect has been reported for *B. cenocepacia* during adaptation to chronic infection, in which a progressive decrease in cell length and conversion from rod to cocci form was observed (Hassan et al. 2019).

### Bacterial cell lifestyle modifications

Biofilm formation in bacteria is mediated by the second messenger bis-(3′–5′)-cyclic dimeric guanosine monophosphate (c-di-GMP) (Jenal et al. 2017). Diverse environmental stressful conditions (e.g. aromatic compounds, oxidative stress, nutrient scarcity, heavy metals, and antimicrobials) in bacterial cells change intracellular levels of c-di-GMP, which is sensed by effectors that activate a stress response (Álviz-Gazitúa et al. 2019, Wang et al. 2023). Potential stress responses are biofilm formation, activation of oxidative stress response, stimulation of antimicrobial or heavy metal resistance, PHA degradation, and regulation of catabolic genes.

A significant decrease in biofilm formation in *P. xenovorans* has been observed during growth on the aromatic compound *p*-cymene compared to glucose-grown cells (Agulló et al. 2017). Proteomic analysis of *P. xenovorans* cells during growth on *p-*cymene showed a stress response and the downregulation of the diguanylate cyclase gene-encoded protein (BxeB2035). The decrease in diguanylate cyclase correlates with an increase in the MotB protein, which is associated with the flagellar motor and cell motility. Stress also reduced biofilm levels in *C. metallidurans* CH34, a member of the Burkholderiales order. In response to cadmium, the aromatic-degrading strain *C. metallidurans* CH34 inhibits the initiation of a biofilm lifestyle that involves a decrease in c-di-GMP levels, mediated by a novel metal-regulated phosphodiesterase (*mrp* gene), which is upregulated in the presence of cadmium. This study established a key connection between the adaptation to heavy metals and a second messenger, which is involved in bacterial lifestyle and many other processes, including electricity production (Alviz-Gazitua et al. 2019, 2022). In contrast, toluene caused in *P. putida* KT2440 a downregulation of the genes encoding proteins involved in flagellar biosynthesis (FlgE and Hag) (Domínguez-Cuevas et al. 2006).

## Accelerated degradation of aromatic compounds by stress-adapted bacteria

To establish optimized biodegradation for aromatic compounds, it is crucial to maintain the fitness of the cells subjected to stressful conditions and to overcome dead-end steps in the catabolic process to avoid the accumulation of toxic metabolites. Maintenance of redox balance within the cell through biotechnological strategies can counteract the oxidative stress generated during aromatic metabolism.

Notably, Ponce et al. (2011) showed that antioxidant compounds such as α-tocopherol stimulate degradation of PCBs by *P. xenovorans* in polluted water and soil. In presence of α-tocopherol, growth and cell integrity of *P. xenovorans* exposed to 4-chlorobiphenyl is less affected, which suggests a protective effect of the antioxidant molecule on the cell membranes (Ponce et al. 2011). Recent studies showed that the overexpression of the long-chain flavodoxin FldX1 improves *P. xenovorans* degradation of aromatic compounds. Proteomic analysis showed a decreased oxidative stress response in FldX1-overexpressing cells (Rodríguez-Castro et al. 2019, 2024). Increased growth and degradation of 4-hydroxyphenylacetate by *P. xenovorans* recombinant cells compared with the control strain were observed (Rodríguez-Castro et al. 2024). Moreover, 4-hydroxyphenylacetate was completely degraded after 3 days in soils bioaugmented with the recombinant *P. xenovorans* strain (Rodríguez-Castro et al. 2024). These results suggest that counteracting stress response may enhance the biodegradation of other aromatic and toxic compounds. For example, metabolic engineering successfully overcame a metabolic dead-end step, improving bioremediation of PCBs (Saavedra et al. 2010, Seeger et al. 2011). The recombinant Burkholderiales strain *C. necator* JMS34 bearing the *bph* locus from *P. xenovorans* mineralizes 3-chlorobiphenyl, 4-chlorobiphenyl, 2,4′-chlorobiphenyl, and 3,5-chlorobiphenyl, without accumulation of chlorobenzoates (Saavedra et al. 2010, Seeger et al. 2011). Within the Burkholderiales order, *Comamonas testosteroni* has been reported as an emerging cellular chassis for bioremediation strategies (Tang et al. 2018)

The overexpression of antioxidant enzymes, specifically ferredoxin-NADP^+^ reductase (Fpr) and SOD, significantly improved the growth and naphthalene degradation rates in recombinant *Pseudomonas* sp. As1 compared to the wild-type strain. This indicates that oxidative stress during naphthalene metabolism may be mitigated by these enzymes. These results suggest that the overexpression of antioxidant enzymes not only helps in oxidative stress management but also contributes to enhanced naphthalene degradation, which is crucial for bioremediation strategies (Kang et al. 2007).

Recombinant *P. putida* KT2440 carrying a naphthalene catabolic plasmid showed improved rhizoremediation performance (Fernández et al. 2012). The NAH7 catabolic plasmid enables *P. putida* KT2440 to degrade naphthalene while mitigating the cellular stress associated with this toxic compound. Strain KT2440R(NAH7) activates a broad stress response in the presence of naphthalene, improving tolerance to naphthalene-induced stress, biomass formation, and, therefore, rhizoremediation.

The ability of *P. putida* strains to resist environmental stressors such as high salinity, temperature variations, and toxic substrates converts them into valuable candidates for bioremediation applications. The metabolic versatility allows the biodegradation of a variety of pollutants (e.g. aromatic and aliphatic hydrocarbons) under challenging conditions. However, genetic modifications that enhance specific metabolic pathways can also contribute to stress tolerance. For example, enhancing the enzymatic activities of the ED and PP pathways in *P. putida* favors NADPH generation and can lead to more efficient degradation of pollutants, thereby enhancing bioremediation processes (Nikel et al. 2021). The introduction of genes that improve redox balance or enhance the degradation of toxic compounds has been proposed to support bacteria surviving and thriving in polluted environments (Sánchez-Pascuala et al. 2018, Martínez-García and de Lorenzo 2024).

The application of synthetic degrading consortia for bioremediation also contributes to the complete mineralization of polycyclic aromatic hydrocarbons and a reduction of stress associated with toxic metabolites (Laothamteep et al. 2021, Nieto et al. 2023). Polycyclic aromatic hydrocarbon degradation was enhanced by a synthetic consortium composed of *Burkholderia* and *Sphingomonas* strains (Nieto et al. 2023). Interestingly, metaproteomic studies showed downregulation of stress-related proteins, indicating that the synergistic relationship of both strains toward complete PAH catabolism also decreases ROS and mitigates stress, thus increasing degradation efficiency (Nieto et al. 2023). In addition, a coculture of *Pseudomonas reinekei* and *Achromobacter xylosidans* showed no accumulation of toxic intermediates, contributing to a downregulation of the stress response, which improved bacterial fitness (Bobadilla-Fazzini et al. 2010).

While extensively studied models such as *P. putida* KT2440 have provided significant insights into stress response affecting biodegradation performance, degrading strains of the Burkholderiales order have gained less attention; this gap in knowledge needs further exploration. Particularly, novel bioremediation approaches addressing stress response to toxic compounds are crucial for the effective application and performance of Burkholderiales strains.

## Concluding remarks

This review highlights the complex mechanisms employed by Burkholderiales and environmental bacteria, with particular emphasis on *P. xenovorans*, to address the challenges associated with the degradation of aromatic compounds and abiotic stressors. The outstanding capability of *P. xenovorans* to degrade toxic aromatic compounds and their metabolites is linked to its stress response mechanisms, which include general stress and antioxidant adaptive responses. In the general stress response, molecular chaperones play a crucial role in maintaining a healthy proteome upon exposure to a variety of stressors, including stress related to aromatic compound degradation and abiotic stresses. In the antioxidant response, the OxyR and SoxR transcriptional regulators play a key role in orchestrating specific responses against oxidative stress caused by the degradation of aromatic compounds and ROS.

Despite several stress studies that have been conducted in aromatic-degrading Burkholderiales strains in the last three decades, diverse questions remain unanswered. For example, we need to deepen in diverse aspects of Burkholderiales strains: (i) the mechanisms that explain why diverse Burkholderiales strains showed superior resistance to stress caused by the degradation of toxic aromatic compounds compared to strains from other taxa, such as Actinomycetota and Bacillota; (ii) the hierarchy of the stress response regulation network, (iii) the mechanisms of antioxidant proteins (e.g. SOD), antioxidant molecules (e.g. glutathione and ß-hydroxy-butyrate) and chaperones to maintain the cellular redox balance; (iv) the response mechanisms to abiotic stresses such as salinity, nutrient scarcity, presence of heavy metals, cold and heat shock; (v) the physiological responses of the membrane, morphology, and lifestyle to aromatic compounds, and (vi) the role of the second messengers in stress responses that has been only partially characterized in a few strains.

This knowledge will pave the way for the degradation performance optimization of *P. xenovorans* and other related environmental strains and the development of more robust and efficient bacteria for bioremediation applications, especially in ecosystems with contamination and hostile environmental conditions, such as salinity and nutrient scarcity.

In this review, genomic analyses unveiled for the first time *P. xenovorans* adaptive strategies, including regulating proteostasis networks, modulation of carbon metabolism, and synthesizing osmoprotectants to restore osmotic balance. However, future experimental studies should aim to deepen the understanding of stress response mechanisms in *P. xenovorans* and other Burkholderiales species during aromatic compound degradation under environmental stressors. Expanding these studies to diverse environmental conditions and other environmentally relevant pollutants can provide insights into specific molecular pathways and regulatory networks that drive bacterial adaptation.

Synthetic biology remains underdeveloped in Burkholderiales. However, biotechnological tools such as CRISPR-based genome editing and omics technologies will enable the engineering of more robust strains. Diverse Burkholderiales strains exhibit promising biodegradation capabilities, which need further exploration for large-scale bioremediation and biotransformation applications. Such bacterial strains may serve as effective bioremediation catalysts, improving pollutant degradation in challenging environmental scenarios.

## Supplementary Material

fuaf021_Supplemental_File
